# Synthetic approaches to potent heterocyclic inhibitors of tuberculosis: A decade review

**DOI:** 10.3389/fphar.2022.1021216

**Published:** 2022-10-31

**Authors:** Upala Dasmahapatra, Kaushik Chanda

**Affiliations:** Department of Chemistry, School of Advanced Sciences, Vellore Institute of Technology, Vellore, India

**Keywords:** tuberculosis, heterocycles, synthesis, *in vitro*, *Mycobacterium tuberculosis*, docking

## Abstract

Tuberculosis (TB) continues to be a significant global health concern with about 1.5 million deaths annually. Despite efforts to develop more efficient vaccines, reliable diagnostics, and chemotherapeutics, tuberculosis has become a concern to world health due to HIV, the rapid growth of bacteria that are resistant to treatment, and the recently introduced COVID-19 pandemic. As is well known, advances in synthetic organic chemistry have historically enabled the production of important life-saving medications that have had a tremendous impact on patients’ lives and health all over the world. Small-molecule research as a novel chemical entity for a specific disease target offers in-depth knowledge and potential therapeutic targets. In this viewpoint, we concentrated on the synthesis of a number of heterocycles reported in the previous decade and the screening of their inhibitory action against diverse strains of *Mycobacterium tuberculosis*. These findings offer specific details on the structure-based activity of several heterocyclic scaffolds backed by their *in vitro* tests as a promising class of antitubercular medicines, which will be further useful to build effective treatments to prevent this terrible illness.

## Graphical Abstract

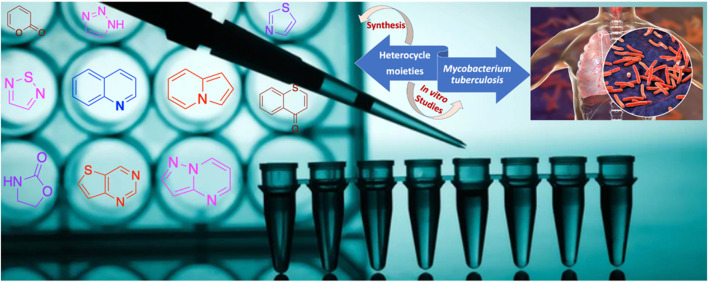

## Introduction

The year 2020 gave us glimpses of what happens in reality when untreatable neglected infectious diseases spread freely. There are several issues such as healthcare, hospital saturation, mammoth lethality, economic burden, and political mistrust that accompanied this huge global pandemic (Yamey et al., 2017; Global Preparedness Monitoring Board, 2019). But to our knowledge, COVID-19 is not the only infectious disease with an epidemic potential. Tuberculosis (TB) is one of the most lethal infectious diseases that man has ever encountered. Since ancient times, tuberculosis has plagued the world, and a tuberculosis diagnosis was regarded as a death sentence. Tuberculosis (TB) can be caused by multiple *Mycobacterium tuberculosis* (*M.tb*) complexes such as *Mycobacterium pinnipedii*, *Mycobacterium microti*, *Mycobacterium canettii*, *Mycobacterium bovis*, *Mycobacterium africanum*, and *Mycobacterium caprae* (Furin et al., 2019; Torres Ortiz et al., 2021). The occurrence of multidrug and a wide range of drug-resistant strains of *Mycobacterium tuberculosis* (*M.tb*) is a growing concern that needs to be addressed (Zumla et al., 2013). Unlike other microorganisms, *Mycobacterium tuberculosis* has infected approximately 1.7 billion people around the world, accounting for more than 20% of the global population. This infectious disease, which is classified as a pandemic, sickens nearly 10 million people each year. According to WHO findings, tuberculosis (TB) nearly claimed the lives of 1.5 million people in 2020 (World Health Organization, 2021a). Due to the COVID-19 pandemic, there was a reduced access to its diagnosis, treatment, and the providing of essential services. Due to this, it has caused the mortality of already affected people and, simultaneously, it was spread among other healthy individuals. Tuberculosis mostly affects the lungs, which can spread from person to person through air, and active pulmonary TB patients are the main source of infection. Despite the fact that a large proportion of infected people may clear the latent infection with time, tuberculosis is one of the World Health Organization’s top 10 causes of death (Holzheimer et al., 2021; World Health Organization, 2020; Fu et al., 2021). One year is the typical treatment time for antitubercular drugs against drug-susceptible tuberculosis, whereas treatment for drug-resistant tuberculosis can take years. In both cases, a lengthy course of antibiotics is required, and adherence is essential for success (Gandhi et al., 2010). The bacterium is mostly shielded from other immune reactions while gaining access to host resources, and after antibiotic treatment, it frequently enters a dormant state inside the human host (Kiran et al., 2016). With the advent of HIV, the situation indirectly increased the severity of the disease; 1.3 million deaths were reported in 2020 among HIV-negative cases with additional deaths of 2.1 million in HIV-positive people. A total of 9.9 million cases were reported in 2020. South-East Asia and Africa are mostly prone to this disease with an account for 85% of total TB deaths in 2020. This trend was observed at all levels: global, regional, and country. The pandemic has inverted years of improvement and research on TB due to the feebleness of governing authorities in its diagnosis. It is estimated that TB would have caused an enormous number of deaths worldwide after the COVID-19 pandemic in 2020 (Ryckman et al., 2022). It affects people of all ages. However, men are more affected than women and children. Tuberculosis co-infected with HIV is more reported in the African region (World Health Organization, 2021b; Moyo et al., 2022).

Globally, the brighter part in tuberculosis elimination is that countries like the United States and European region have low incidence, whereas countries like India, China, and Indonesia constitute a major part of affected cases. For its treatment, the WHO divided drug-resistant TB into five categories. While many people have latent TB infections that are asymptomatic, active pulmonary TB patients are the main source of infection since it primarily affects the lungs and can spread from person to person through air.

Extensive research on tuberculosis involved many routes to find a potent molecule for its treatment. Many efforts have been made in recent years to develop an effective TB drug pipeline with currently positive results (Evans and Mizrahi, 2018; Libardo et al., 2018; Conradie et al., 2020). Several heterocyclic molecules (Yan and Ma, 2012), peptides (Usmani et al., 2018), and natural products (Igarashi et al., 2017) are also evaluated in drug discovery programs as antitubercular agents. Both medicinal chemistry targets and pharmaceuticals that are now on the market typically use heterocyclic scaffolds as their chemical building blocks. The extreme predominance of oxygen, sulfur, and particularly nitrogen-containing rings in pharmacological compounds is clear. Considering that heterocycles are the fundamental components of a variety of natural compounds, medicinal chemistry studies frequently center on mimicking similar structural patterns. Heterocyclic molecules are both biologically active and toxic, depending on various reasons such as concentration, metabolites formed, and half-life of the moiety. The major reason that controls all of the factors is molecular weight. Although the concentration of the lead molecule during biological studies can be tuned, a number of metabolites formed after phase-I and phase-II need to be checked at the desk (Bramhankar and Jaiswal, 1995).

The latest advancements in organic chemistry and strategic route reconnaissance, which are strongly supported by novel synthetic techniques, catalysis, machine learning, and high-throughput experimental technologies, are also thought to be significant for drug development. Within the framework of the research carried out by our group for the development of novel heterocyclic scaffolds (Rao and Chanda, 2022; Panchangam et al., 2021; Padmaja et al., 2019; Panchangam et al., 2019), we discuss various available heterocyclic moieties and drug candidates identified by a target-based approach against tuberculosis diseases. We go in depth on many synthetic techniques that start with distinct synthetic sequences and the ensuing antitubercular activity in this article. Last but not least, combining all synthetic modifications and antitubercular activity will provide essential solutions to the current difficulties in discovering novel antitubercular drugs and will also support knowledge about the current status of antitubercular drug discovery. This review study will also help medicinal and synthetic chemists work together to speed up the development of new antitubercular drugs.

## Synthesis and antitubercular activity of heterocyclic moieties

**FIGURE 1:**
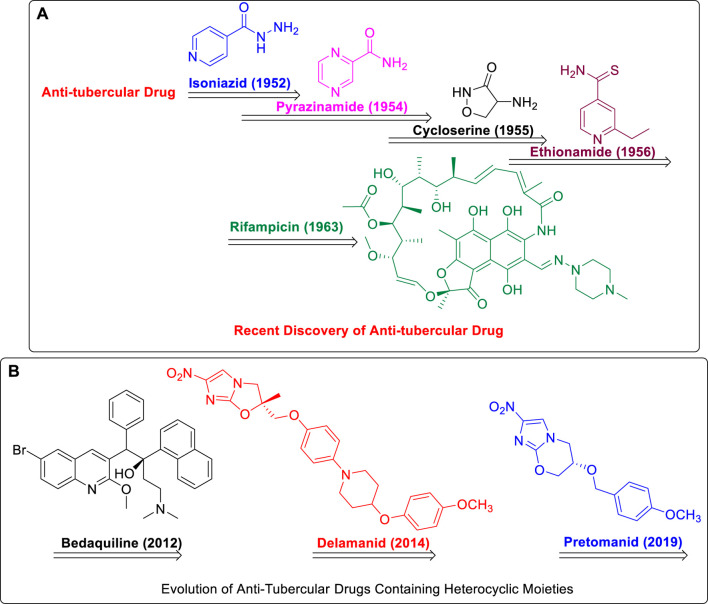
**(A)** Recent Discovery of Anti-Tubercular Drugs. **(B)** Evolution of Anti-Tubercular Drugs Containing Heterocyclic Moieties.

Streptomycin, the first anti-TB drug found after penicillin, was developed in 1943 using *Streptomyces griseus*. Isoniazid, pyrazinamide, cycloserine, ethionamide, and rifampin are the most popular heterocyclic molecules developed until early 1960 as anti-TB drugs (Figure 1) (Chauhan et al., 2010). The treatment for TB has not changed much since then, and long-term use of these drugs is associated with substantial toxic side effects and treatment resistance. On 28th December 2012, the FDA approval of bedaquiline, that is, the first antituberculosis drug to be licensed in more than 40 years, was an important step in this direction. Subsequently, in 2014, delamanid was approved by the European Medicines Agency (EMA), and recently, pretomanid was approved by FDA strictly for use in combination with bedaquiline and linezolid to treat severe drug-resistant tuberculosis (Ryan and Lo, 2014; Keam, 2019; Lubanyana et al., 2020; Guo et al., 2021; Aono et al., 2022).

This year, Fernandes et al. have extensively reviewed the *in vivo* efficacy of antitubercular drug candidates against *Mycobacterium tuberculosis.* The report covered only those molecules that entered into clinical trials in the last 6 years (Fernandes et al., 2022). Compared to the recent report in 2022 (Fernandes et al., 2022), where there were no synthetic methodologies described, we have made an effort to highlight the most noteworthy examples of heterocyclic moieties reported in the last decade, highlighting various strategies to develop potential novel compounds with antitubercular properties. The potential antitubercular action of hybrid compounds incorporating isoniazid with various heterocyclic scaffolds, on the other hand, was an intriguing finding in recent studies (Reis et al., 2019; Johansen et al., 2021; Alcaraz et al., 2022).

**SCHEME 1:**
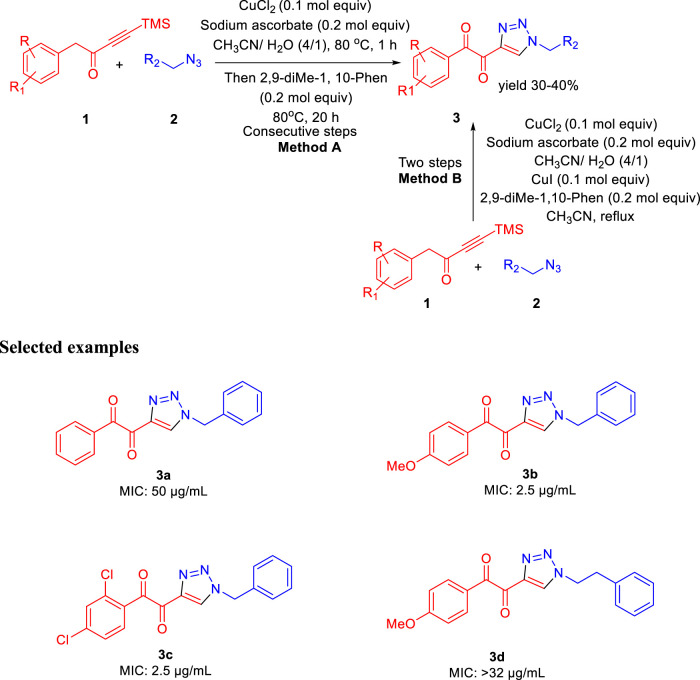
Synthesis of α,β-diketotriazoles from both TMS-ynones and azides *via* the click pathway.

In 2013, Baltas *et al.* reported the synthesis of α,β-diketotriazoles and investigated the potential biological activity against *Mycobacterium tuberculosis* (Menendez et al., 2013). According to Scheme 1, TMS-ynones 1 and azide derivatives 2 were reacted together to produce α,β-ketotriazoles in the presence of CuCl_2_ and sodium ascorbate using a CH_3_CN-H_2_O mixture as solvent. Subsequently, α,β-diketotriazole derivatives 3 were synthesized by two slightly different protocols. α,β-diketotriazole derivatives 3 were synthesized from the α-ketotriazoles using CuI or CuCl_2_ under reflux conditions and CH_3_CN–H_2_O mixture as solvent at 80°C for 1 h followed by the addition of 2,9-dimethyl-1,10-phenanthroline at the same temperature for 20 h (method A). The one-pot method B (two-step processes) was performed by TMS deprotection, followed by a 1,3-dipolar cycloaddition reaction between azide **2** and deprotected ynone **1** at 80°C using the CH_3_CN-H_2_O mixture as solvent. Finally, the α,β-diketotriazole derivatives 3 were obtained in moderate to excellent yields by the addition of CuI and 2,9-diMe-1,10-Phen under reflux conditions (Menendez et al., 2011; Menendez et al., 2012). On the most researched H37Rv strains of *M. tuberculosis* with a smooth colony shape, all produced α-ketotriazoles, and the corresponding α,β-diketotriazoles have been examined by their minimum inhibitory concentration (MIC). The attenuated tubercle *bacillus M. tb* H37Ra is closely linked to the virulent kind strain *M. tb* H37Rv. The differences between *Mycobacterium tuberculosis* H37Ra and H37Rv’s membranes and carrier proteins may have an impact on how effective an antibiotic treatment is. Predominantly, among the synthesized library, compounds **3b** and **3c** have shown 2.5 μg/ml MIC value with no cytotoxicity toward the HCT116 and GM637 human cells.

**SCHEME 2:**
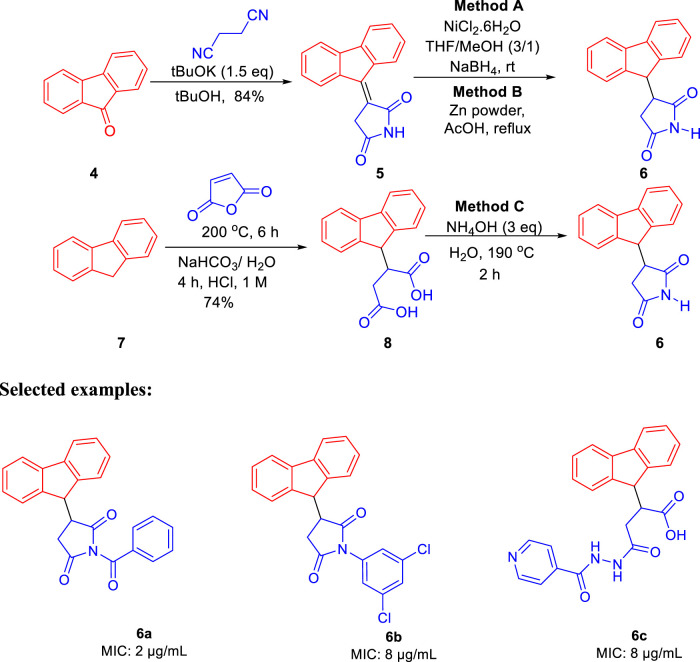
Synthesis of succinimides by multiple methods.

**FIGURE 2:**
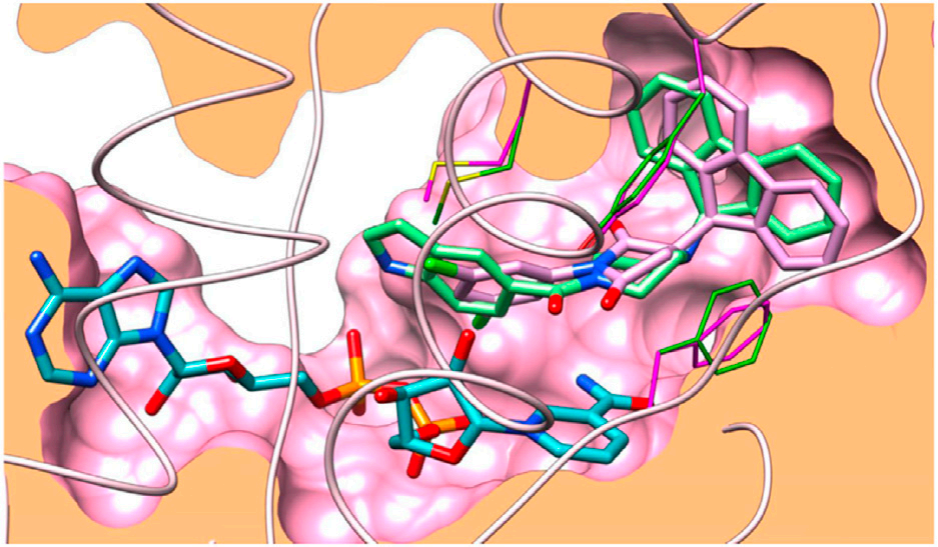
Docking binding of **6b** with InhA. Reproduced from Baltas et al., 2013, with permission from Elsevier, Copyright 2013.

The same group further developed 3-(9H-fluoren-9-yl)pyrrolidine-2,5-dione derivatives by three different pathways (**methods A, B,** and **C**) and evaluated their efficacies against *M. tuberculosis* in the same year (Matviiuk et al., 2013). In **method A**, the fluorenone 4 was condensed with succinonitrile under basic conditions resulting in 3-(9H-fluoren-9-ylidene)pyrrolidine-2,5-dione followed by the reduction of the double bond using NiCl_2_ and NaBH_4_, which led to the formation of compound **6** with 40% yield, whereas in **method B**, zinc powder and acetic acid were used under reflux conditions to gave a yield of 97% of compound **6** (López-Rodríguez et al., 1999; Ballini et al., 2003; Cheng et al., 2008). Compound **8** was prepared by reacting maleic anhydride and fluorene at 200°C followed by basification with ammonium hydroxide at 190°C resulting in the formation of compound **6** with a 73% yield in **method C** (Scheme 2) (Bergmann and Orchin, 1949). Compound **6a** strongly inhibited the growth of *M. tuberculosis* with an MIC value of 2 μg/ml toward H37Rv strain of *M. tb*. On other hand, compound **6b** having a 3,5-dichloromethyl substituent on nitrogen atom exhibited good inhibition with 8 μg/ml and was further chosen for molecular docking studies for being the best InhA inhibitor among the synthesized derivatives (Figure 2).

Like the GEQ inhibitor, compound **6b** binds to InhA’s binding pocket following the same pattern. Hydrogen bonding with the residue of Tyr158 is predominantly conserved, while other binding site residues (Met161 and Phe149) showed minor deviations.

**SCHEME 3:**
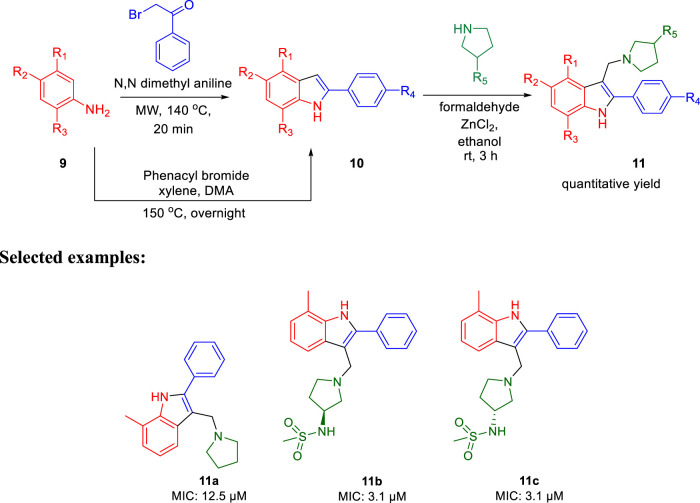
Microwave-assisted synthesis of phenylindoles.

In 2014, Chatterji and co-workers demonstrated the synthetic route to 2-phenylindole along with arylsulfonamide and studied their potency against *M. tuberculosis* (Naik et al., 2014). Aniline derivative 9 was reacted with phenacyl bromide in the presence of *N,N′*-dimethyl aniline under microwave conditions for 20 min at 140°C, resulting in compound **10** (phenylindole), as depicted in Scheme 3. Compound **10** was also synthesized using another protocol, in which aniline derivative 9 was reacted with phenacyl bromide in the presence of xylene, DMA at 150°C for 12 h (Mahboobi et al., 2008; Amblard et al., 2013). Subsequently, 3-alkyl pyrrolidine reacted with compound **10** in the presence of ZnCl_2_ and ethanol at ambient temperature for 3 h to produce the corresponding phenylindole derivative **11** with excellent yield.

**SCHEME 4:**
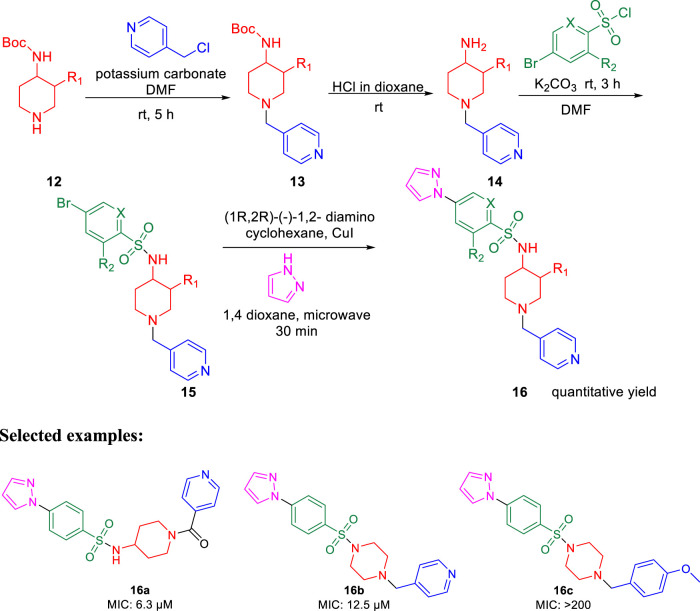
Microwave-assisted synthesis of arysulfonamide derivatives.

Furthermore, arylsufonamides were synthesized from the NHBOC protected 4-aminopiperidine **12** using microwave irradiation. The NHBOC-protected 4-aminopiperidine **12** was reacted with pyridine methyl halide using K_2_CO_3_ as base in DMF solvent at ambient temperature for 5 h. Next, the BOC deprotection was performed in dioxane containing HCl solution at room temperature to obtain compound **14**. Subsequently, the NH-sulfonylation of compound **14** was executed in DMF solution using bromo-substituted sulfonyl chloride at room temperature for 3 h. Compound **15** was then reacted with pyrazoles using (1*R*,2*R*)-(−)-1,2-diaminocyclo-hexane and CuI as catalyst in dioxane solution under microwave conditions for 30 min to obtain the final compound **16** (Scheme 4).

**FIGURE 3:**
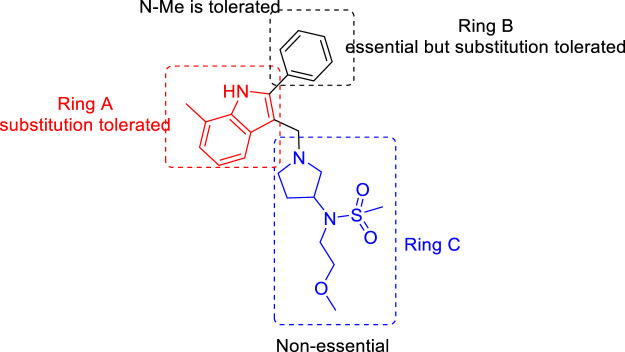
SAR for 2-phenylindoles.

For the 2-phenylindole scaffold, a robust SAR was constructed, which resulted in lead-like structures with good physicochemical attributes. The chemical optimization of 2-phenylindoles has been illustrated; 2-phenylindole was broadly divided into ring A, ring B, and ring C, as depicted in Figure 3. The impact of various groups on ring A also showed that electron-donating substituents such as methyl, methoxy, and isopropyl were allowed at the C-7 position, and ring C is a nonessential part.

**FIGURE 4:**
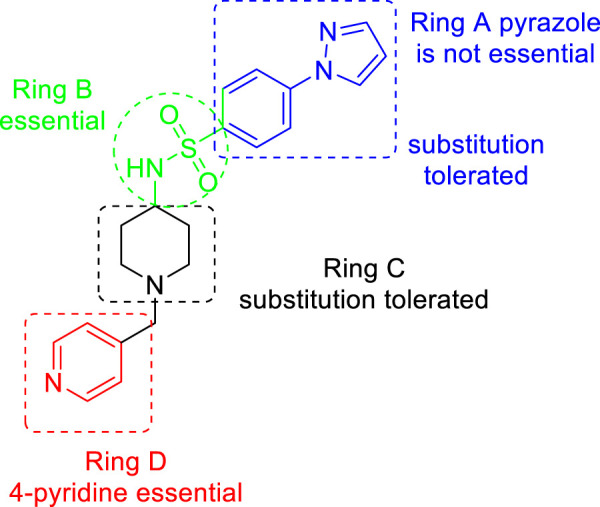
SAR for arylsulfonamides.

Similarly, SAR of arylsulfonamide also suggested (Figure 4) that the presence of ring B is essential, whereas the presence of pyrazole ring A is not essential. The investigation showed that the alteration of the pyrazole moiety (ring A) to oxazole did not change the potency of the compounds as antitubercular agents. It was also found that ring C’s substitution was tolerated, with the indication of necessity of ring D, that is, 4-pyridine. Testing of the synthesized 2-phenylindoles showed the antimicrobial property to H37Rv strain with ∼5–10 ratios of MBC to MIC, whereas the arylsulfonamides displayed ∼1–2 ratios for the same. Among the synthesized phenylindoles, compounds **11b** and **11c** showed strong potency with an MIC value of 3.1 μM. The MIC values for synthesized arylsulfonamides **16a** and **16b** on the other hand were 6.3 and 12.5 μM, respectively.

**SCHEME 5:**
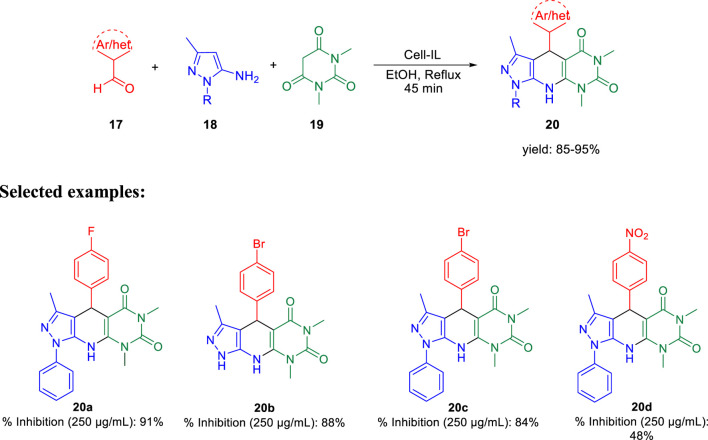
Cellulose-supported ionic liquid-catalyzed synthesis of pyrazole-based pyrido[2,3-d]pyrimidine-diones.

**SCHEME 6:**
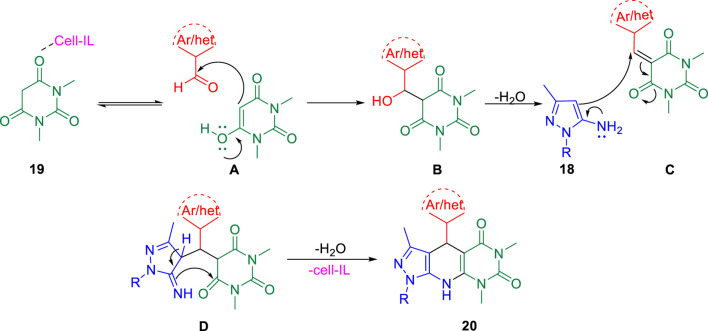
Plausible mechanistic pathway for the synthesis of pyrido[2,3-*d*]pyrimidine-diones.

Further, in the same year, Raval *et al.* accomplished the synthesis of pyrazole-fused pyrido[2,3-d]pyrimidine-dione derivatives and evaluated their biological properties (Satasia et al., 2014). Pyrazole-fused pyrido[2,3-d]pyrimidine-dione derivatives **20** were synthesized by the reaction of aldehyde **17**, aminopyrazoles **18**, and 1,3-dimethylbarbituric acid **19** in the presence of cell-IL as catalyst in ethanol solvent under reflux conditions and resulted in excellent yields (Scheme 5). The synthesis of cellulose-based ionic liquids (cell-ILs) was reported by the same group (Satasia et al., 2013). The solvents were found to have a significant influence on the synthesis of this particular series of compounds. Among the solvents such as acetonitrile, DMSO, methanol, ethanol, water, DMF, and THF, the best results were obtained in ethanol solvent in terms of influencing the reaction and isolating the targeted compound. A plausible mechanism for the synthesis of pyrido[2,3-d]pyrimidine-diones is depicted in Scheme 6. An attempt to perform the reaction without catalyst resulted in a very poor yield of the product. To evaluate the biological activities of this compound against the H37Rv strain of *M. tuberculosis*, *in vitro* antitubercular tests were performed using isoniazid and rifampicin drugs as the standard drugs. Primary screening was executed using the conventional method, that is, Lowenstein–Jensen medium, at 250 and 100 μg/ml, in which compound **20a** unveiled 91% inhibition at a concentration of 250 μg/ml and 88% inhibition at a concentration of 100 μg/ml.

**SCHEME 7:**
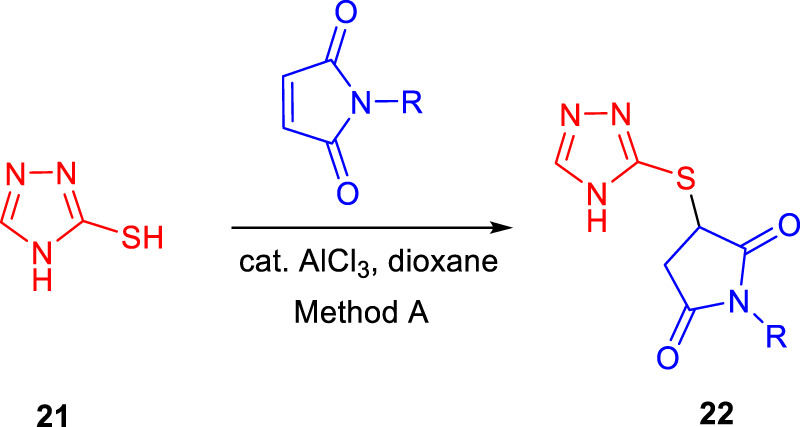
Synthesis of compound **22** from 4*H* to 1,2,4-triazole-3thiole by catalytic conjugate addition.

**SCHEME 8:**
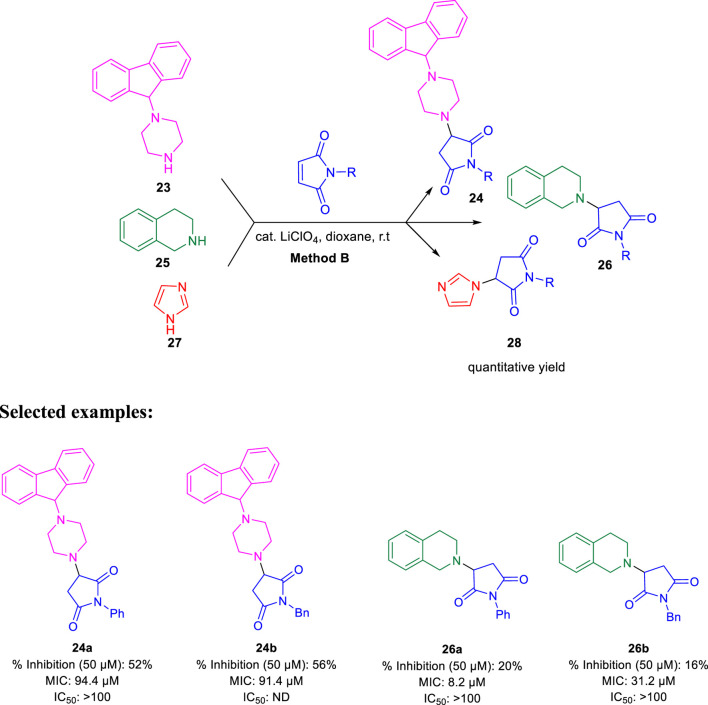
Lithium perchlorate catalyzed synthetic route to C–N Michael adducts.

**SCHEME 9:**
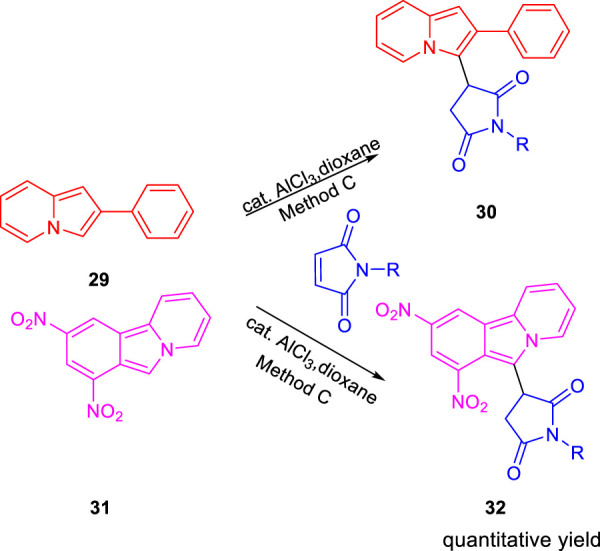
Synthesis of compound **30** and compound **32** by catalytic conjugate addition using N-substituted maleimides.

Subsequently, Baltas and co-workers reported different methods of synthesizing 3-heteroaryl-substituted pyrrolidine-2,5-diones by catalytic Michael reaction and estimated their inhibition against *Mycobacterium tuberculosis* (Matviiuk et al., 2014a). Conjugated Michael addition is a well-studied tool to prepare peptide analogs, antibiotics, and pharmaceutical intermediates along with drugs (Kumagai et al., 2003; Bartoli et al., 2006; Shrestha et al., 2012). Compound **21** with encompassing ambident nucleophile was reacted with maleimide by conjugated Michael addition using a catalytic amount of aluminum chloride to generate compound **22** with high yields by **method A**, as depicted in Scheme 7. In another process (**method B**), Michael addition was performed using a catalytic amount of lithium perchlorate and dioxane at room temperature with good yield, as shown in Scheme 8. With **method C**, to carry out Michael addition between bulky heterocycles and nitrogen-substituted maleimides, various catalysts were used including zinc chloride, titanium tetrachloride, tin chloride, and bismuth trichloride. The screening results indicated that C–C conjugated addition was produced in better yields with anhydrous AlCl_3_ as catalyst, whereas C–N conjugated addition was performed in a better way with a catalytic amount of LiClO_4_ (Scheme 9). The synthesized moieties were tested for *in vitro* efficacies as an inhibitor of InhA at 50 μM and the MIC values toward *M. Tuberculosis* H37Rv strain. GEQ, triclosan, and isoniazid were used as standards for the comparative study toward the H37Rv inhibitor. Among all tested derivatives, compounds **24a** and **24b** demonstrated the finest activity on InhA protein with 52% and 56% inhibition, respectively, at 50 μM. To understand the binding pattern of pyrrolidine-2,5-dione-substituted analogs compared to known nanomolar inhibitors, molecular docking was carried out. *R* and *S* enantiomers of compound **24a** along with GEQ were docked inside the InhA binding site with the help of the calculated algorithm and procedure.

**FIGURE 5:**
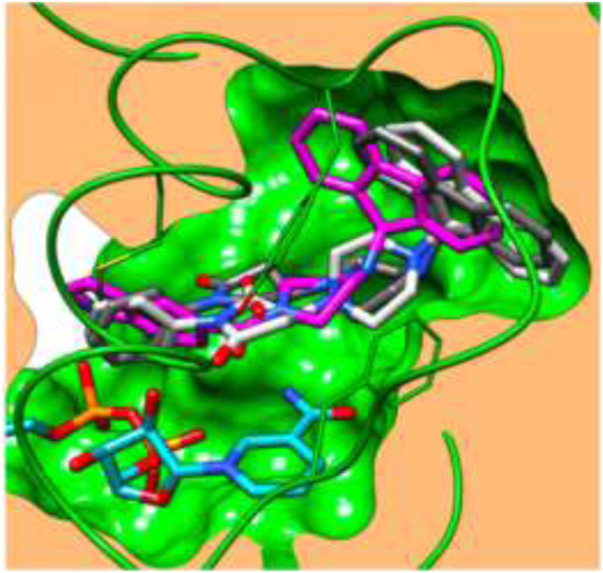
Predicted binding pattern of **24a.** Dark gray represents *R*-enantiomer and light gray represents *S*-enantiomer. Reproduced from Matviiuk et al., 2014a, with permission from Elsevier, Copyright 2014.

Docking (Figure 5) was accomplished with consideration of 11 amino acid residues from the lateral chain, along with a number of cofactor NAD^+^-like traits inside the binding pocket of protein; 0.6 A^o^ was the value of RMSD between crystallographic conformation and the best mode of GEQ docking. From the docking studies and the data of crystallography, it was seen that compound **24a** can be put into the active site by a different geometry. Surprisingly, compound **26a** produced high inhibition while another derivative **26b** showed a reduced activity. Among all of the isoquinoline derivatives, compounds **26a** and **26b** with phenyl and benzyl substituents showed higher inhibition, that is, 20% and 16%, respectively.

**SCHEME 10:**
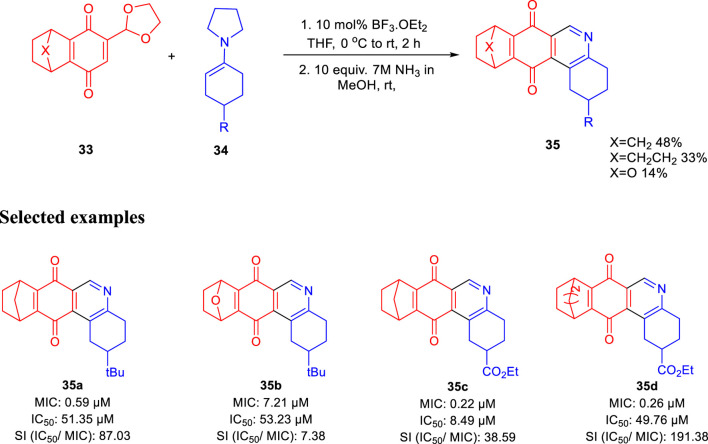
Synthesis of octahydrobenzo[*j*]phenanthridinediones *via* epoxy bridge ring opening.

In the same year, Huygen and group reported 1,2,3,4,8,9,10,11-octahydrobenzo[j]phenanthridine-7,12-diones as an inhibitor against *Mycobacterium tuberculosis* (Cappoen et al., 2014). Condensation of compound **33** with compound **34** using 10 mol% of boron trifluoride and diethyl ether at 0°C and room temperature for 2 h followed by the addition of ammonia in methanol at room temperature obtained resulted in compound **35**, as shown in Scheme 10. Several derivatives of compound **35** were produced with good yield, and antimicrobial activities were tested against luminescent H37Rv strain of *M. tuberculosis* (Forge et al., 2012; Cappoen et al., 2013; Claes et al., 2013). Antitubercular property of these compounds were studied via the depletion of emitted luminescence by an exposure to compound culture. Additionally, the toxicity of these analogs was checked toward the macrophage model of eukaryotic J774 A.1 cells, as these are the main host for tuberculosis infection. Acute toxic concentration (IC_50_) of these compounds was divided by corresponding MIC values to achieve the selectivity index. Compound **35a** demonstrated higher activity (MIC = 0.59 μM) and significantly low toxicity (IC_50_ = 51.35 μg/ml), developing a suitable SI of 87.03. For epoxy-bridged molecule **35b**, the MIC value was reported at 7.21 μM along with a IC_50_ value of 53.23 μg/ml, resulting in 7.38 SI value. Both **35c** and **35d** showed extraordinary MIC values of 0.22 and 0.26 μM, respectively. But due to better IC_50_ values, **35d** resulted in the most favorable SI value, that is, 191.38.

**SCHEME 11:**
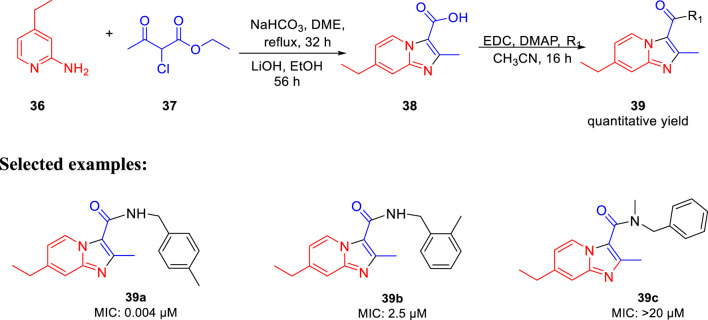
Synthetic route to zolpidem analogs *via* saponification.

Miller *et al.* in 2015 reported the potency of different antitubercular agents toward *M. tb* H_37_Rv strain and substantial drug-resistant *M. tb* strains (Moraski et al., 2016). As depicted in Scheme 11, the group synthesized 4-zolpidem analogs to discover the patterns that impact the biological function. All analogs were converted to form amide bond between the analogous carboxylic acid and amines. As per the anticipation, these rationally designed isomers indicated enhancement contrasted to zolpidem. With an MIC value of 0.004 μM, compound **39a** appeared to be the most potent one. It showed almost similar activity as rifampicin, which is a well-known first-line drug for tuberculosis treatment with an MIC value of 0.1 μM (Cohn et al., 1990). All compounds with insufficient hydrogen bond donors demonstrated weak activity. Considering the effect of stereochemistry, it was found that (*R*)-enantiomer had three times better activity than (*S*)-enantiomer. As a control, PA-824 was used to screen all molecules for *M. tb*-resistant strains (Tover et al., 2000). Surprisingly, compound **39a** showed significant activity with MIC value ˂ 0.03 μM against the most common clinical strains. *In vitro* toxicity testing indicated no significant toxicity to Vero cells (Lilienkampf et al., 2009) or PC-3. However, some of the compounds showed moderate toxicity in the HeLa cell line.

**SCHEME 12:**
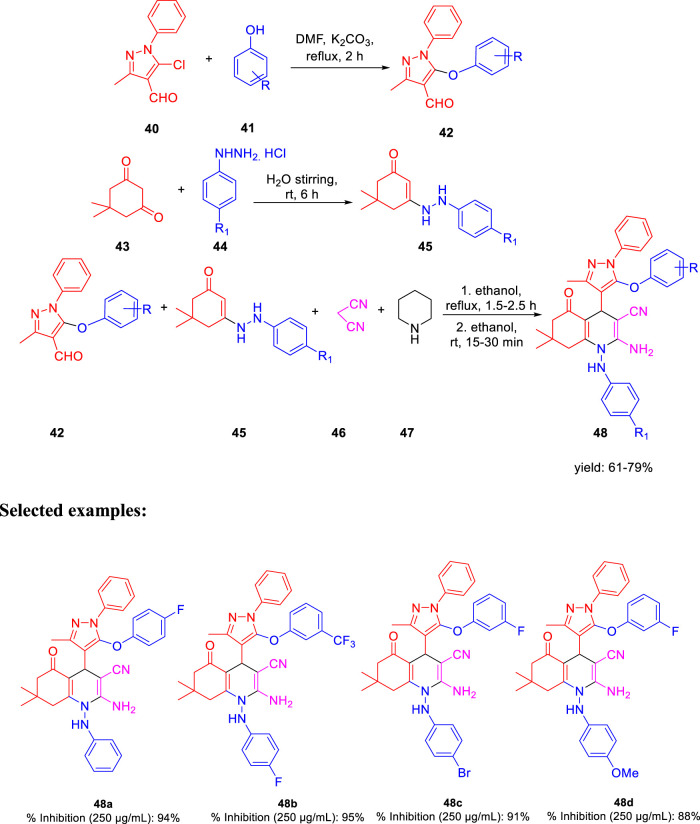
Multistep synthesis of polyhydroquinoline derivatives.

**SCHEME 13:**
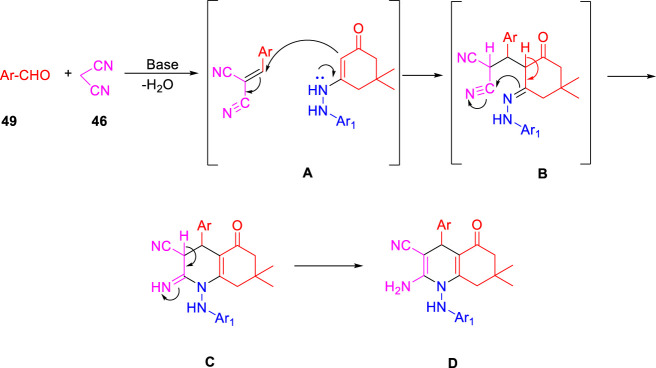
Plausible mechanism for the synthesis of polyhydroquinoline derivatives.

In the subsequent year, Karad and his group established a multistep protocol for the synthesis of a fluorinated 5-aryloxypyrazole nucleus through the cyclocondensation reaction, followed by the investigation of its antitubercular property (Karad et al., 2015). As depicted in Scheme 12, compound **40** and aromatic phenol **41** were refluxed in DMF using K_2_CO_3_ as a base to afford the desired product **42**. Hydrazinoketone **45** was synthesized under aqueous conditions by the reaction of compounds **43** and **44** at room temperature for 6 h. Finally, targeted moiety **48** was obtained by refluxing compounds **42** and **45** *via* cyclocondensation reaction in the presence of malonitrile **46** and piperdine **47**. A plausible mechanism for the synthesis of polyhydroquinoline derivatives is depicted in Scheme 13. *In vitro* studies of antitubercular activity of the synthesized compounds were performed toward the H37Rv strain using Lowenstein–Jensen medium, where rifampicin and isoniazid were taken as standard drugs. Compounds **48a**, **48b**, and **48c** possessed intense activity with 94%, 95%, and 91% inhibition, respectively, at 250 μg/ml. Further interesting facts were obtained when the cytotoxicity was checked for these molecules in the cellular level by the bioassay test of *S. pombe* cells. Variation in the concentration of different types of substituent has a significant effect on the toxicity level. Compound **48b** appeared to be most toxic, whereas compounds **48a** and **48c** are comparatively less toxic.

**SCHEME 14:**
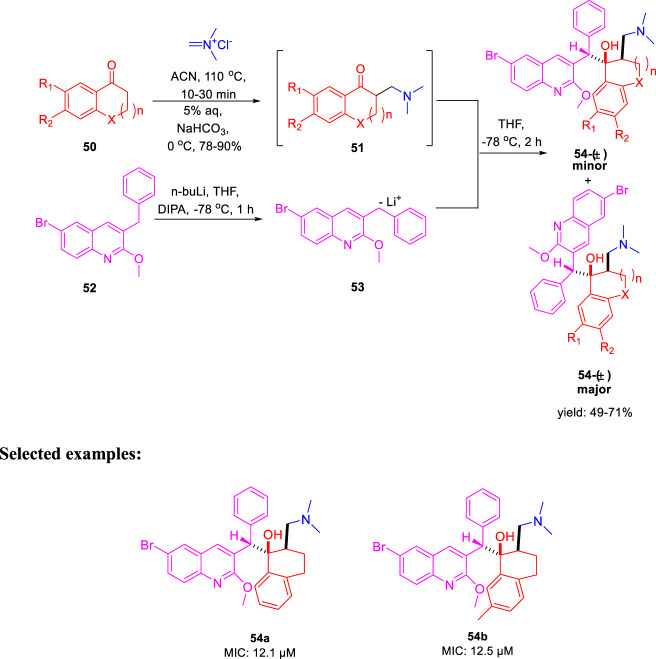
Synthetic route to TMC207 analogs using the Mannich base.

**SCHEME 15:**
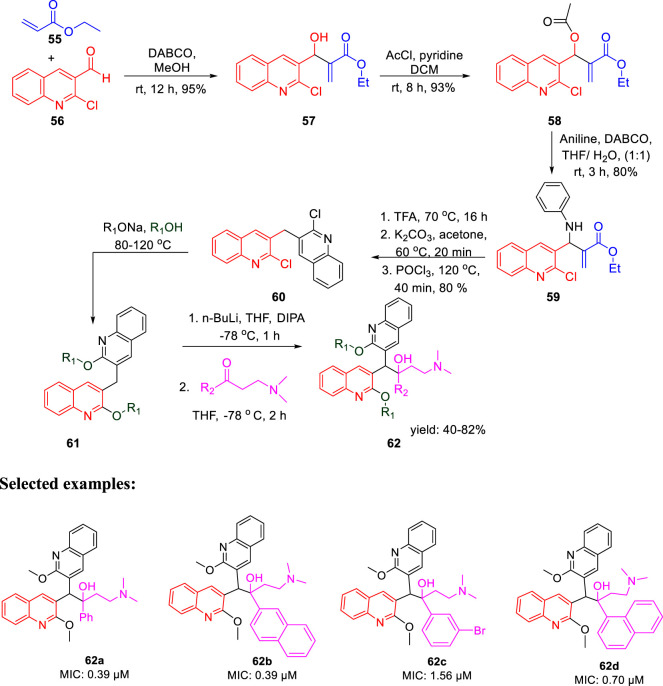
Synthetic route to bisquinoline derivatives using the Mannich base.

In the same year, Kalia *et al.* reported the synthesis of conformationally constrained and bisquinoline analogs of TMC207 and investigated their antitubercular activity (Kalia et al., 2015). Constricted analogs of TMC207 were prepared using benzyl quinoline anion with proper cyclic ketone analogs of the Mannich base as reported earlier (Guillemont et al., 2011). A diastereomeric combination of two conformationally restricted diarylquinolines **54** with five-, six-, and seven-membered rings were synthesized by treating the freshly produced Mannich base **51** with anion **53** (Scheme 14). Subsequently, the bisquinoline analogs of TMC207 were achieved by the reaction of the relevant Mannich bases with the dialkoxybisquinoline anions, which were produced by the treatment of several alkoxides with dichlorobisquinolines. An earlier work that used the Baylis–Hillman adducts to produce quinoline derivatives served as the model to obtain compound **60**, which further reacted with sodium alkoxide under reflux conditions to produce bisalkoxyquinolines **61** (Pathak et al., 2007). In the last step, deprotonation of compound **61** at −78°C, followed by the addition of the Mannich base, led to the formation of compound **62** (Scheme 15). For evaluation of TMC207 analogs as antitubercular agents, BACTEC assay was used on H37Rv *M. tb* strain. It has been found that compound **54a** has the lowest MIC value of 12.1 μM, whereas compound **54b** demonstrated a value of 12.5 μM. A comparison study indicated that bisquinoline analogs of TMC207 have much higher activity than the conformationally constrained molecules.

**FIGURE 6:**
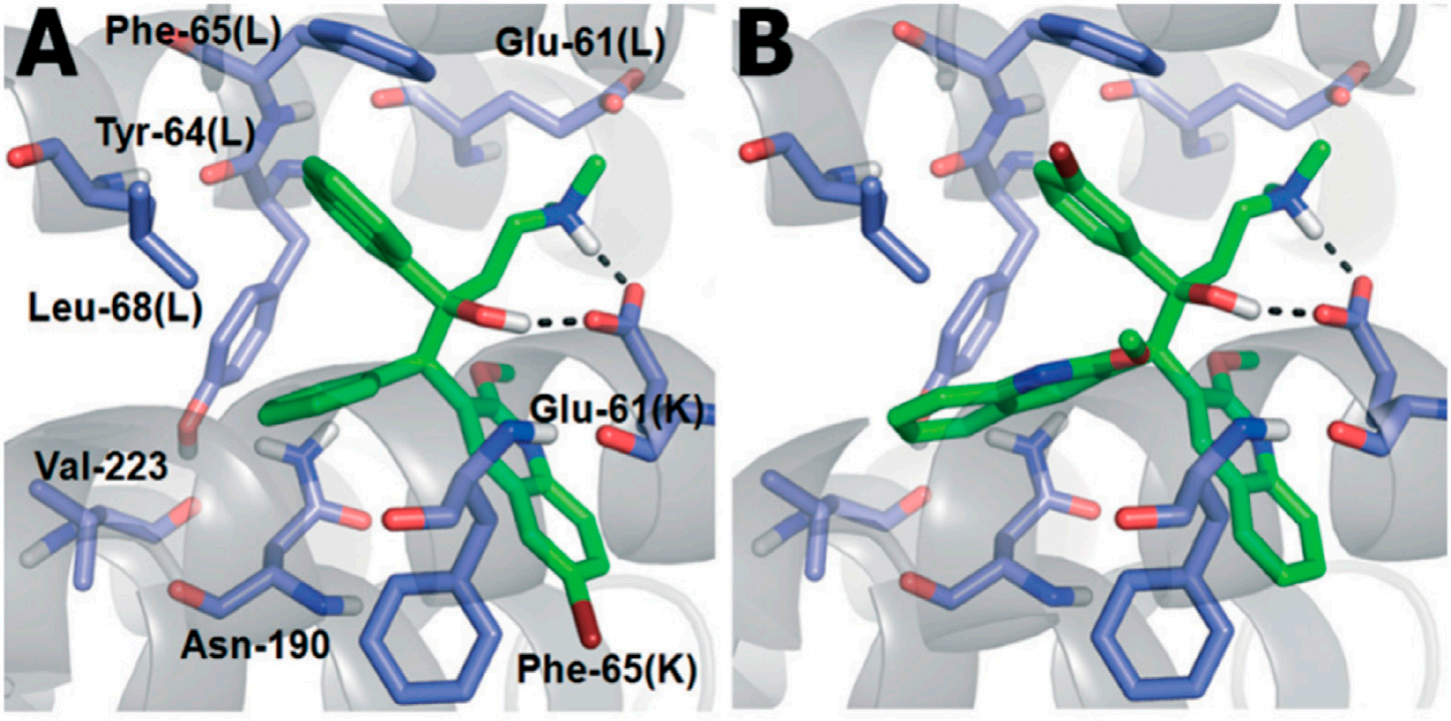
**(A)** Binding mode of TMC207; **(B)** Binding mode of **62C**. Reproduced from Kalia et al., 2015, with permission from Royal Society of Chemistry, Copyright 2015.

There were six compounds with MIC values under 2 μM among the bisquinoline analogs, including compounds **62a** and **62b** (MIC: 0.39 μM). The methoxy group on the second quinoline ring of these molecules appears to be an important factor in their activity. Later on, the cytotoxicity of these compounds was determined toward the macrophages derived from the mouse bone marrow and also toward the Vero cells. Docking studies highlighted the crucial role of both hydrophobic and electrostatic interactions for the stabilization of these moieties to bind into the active site of ATP synthase enzyme, resulting in potent enzyme inhibition (Figure 6).

**SCHEME 16:**
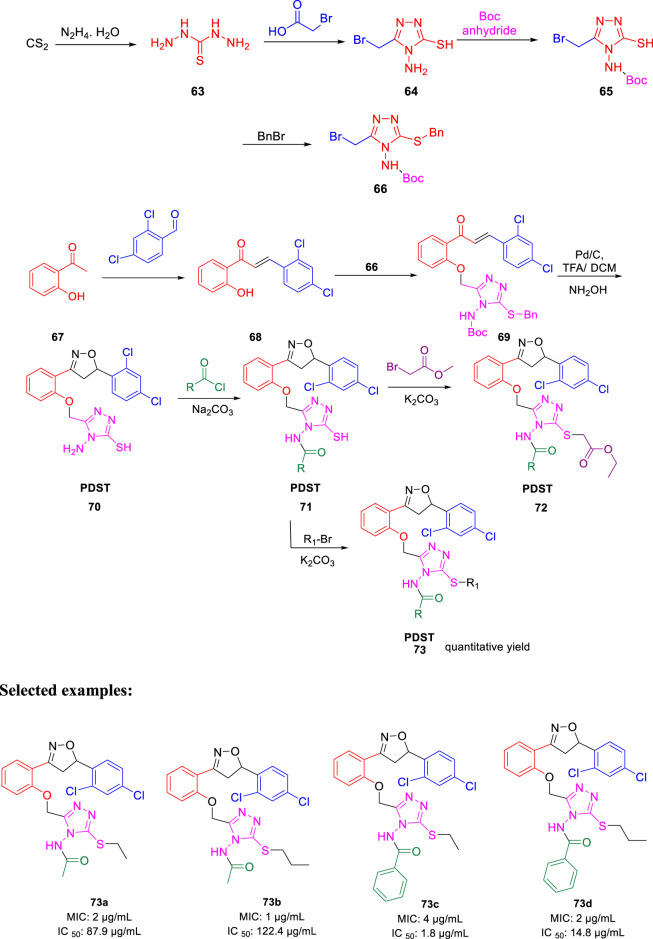
Multistep synthetic route to PDST.

In 2016, Thore *et al.* reported the synthesis of hybrid triazoles and evaluated their potency as dual inhibitors of growth and efflux inhibition in *M. tuberculosis* (Dixit et al., 2016). Over the years, fused and linked triazoles have emerged as a popular antitubercular agent (Shiradkar et al., 2007a; Shiradkar et al., 2007b; Gill et al., 2008; Jadhav et al., 2009). As presented in Scheme 16, hydrazine compound **63** reacted with bromoacetic acid followed by the Boc protection led to the formation of compound **65**. In the next step, the addition of benzyl bromide to compound **65** afforded the protected triazole **66**. Subsequently, compound **67** was treated with 2,4- dichlorobenzaldehyde to achieve intermediate **68**, which further reacted with compound **66** and produced triazolyl-chalocones **69**. Cyclization of the triazolyl-chalocones with hydroxylamine followed by deprotection of amino and thio functionalities resulted in the formation of targeted PDST derivatives. The potency of these hybrid molecules as growth and efflux inhibitor (TB-GEI) toward *M. smegmatis* mc (b) and H37Rv strains was determined. It was observed that compounds **73a**, **73b**, **73c**, and **73d** showed promising MIC values of 2, 1, 4, and 2 μg/ml, respectively. Later, the potency of these hybrid molecules to inhibit efflux of ethyl bromide from *Mycobacterium smegmatis* mc (b) cells was tested *via* real-time fluorometry. Compounds **73a** and **73b** were found to be harmless toward human macrophages with IC_50_ values 87.9 and 122.4 μg/ml, respectively. It was found that some of the synthesized compounds were toxic with IC_50_ value as low as 5.5 μg/ml. Moreover, compound **73a** demonstrated good synergistic action with RIF and INH, whereas it failed to exhibit potency in the case of EtBr.

**SCHEME 17:**
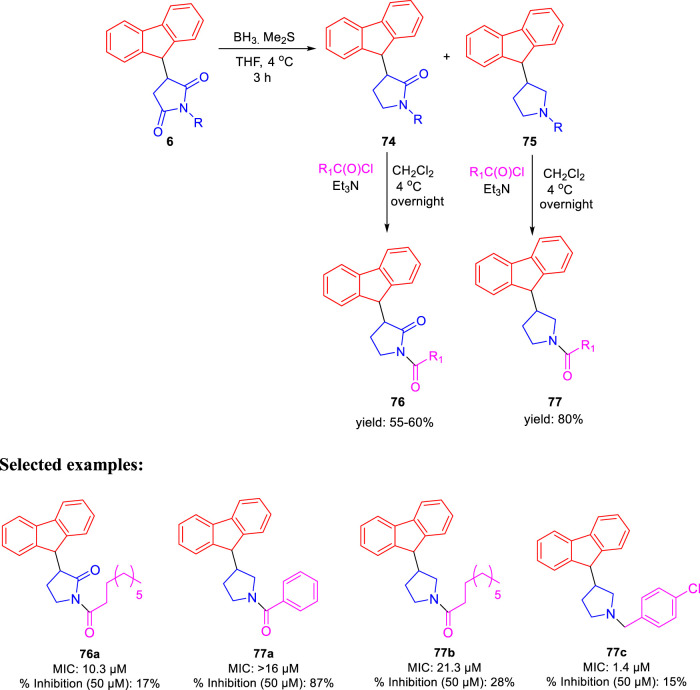
Synthetic route to pyrrolidinone and pyrrolidine derivatives *via* reduction followed by acylation.

**FIGURE 7:**
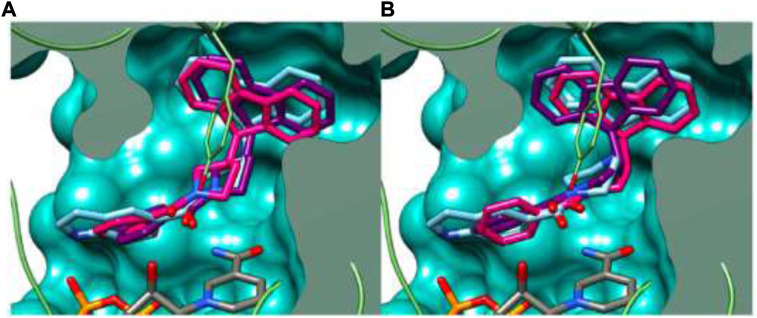
Binding mode of **77a** with InhA; **(A)** (R)-enantiomer and **(B)** (S)-enantiomer. Reproduced from Matviiuk et al., 2016, with permission from Elsevier, Copyright 2016.

In the same year, Baltas *et al.* synthesized pyrrolidinone and pyrrolidine derivatives as the *Mycobacterium tuberculosis* inhibitor (Matviiuk et al., 2016). As depicted in Scheme 17, the succinimide moiety in compound **6** was reduced *via* BH_3_. Me_2_S was used to generate compounds pyrrolidinone **74** and pyrrolidine **75** in the ratio of 1:1 mixture, followed by the acylation reaction using benzoyl chloride (Matviiuk et al., 2014b). To determine the potency of all synthesized compounds toward H37Rv strain, inhibition assay tests were performed at 50 μM. The result indicated that compound **77a** has the best inhibition (87%) toward InhA enzyme, which is selected for docking studies. It is observed from the docking studies that GEQ and compound **77a** (*R* and *S* enantiomers) can adopt a similar conformations and interactions in the active site of InhA (Figure 7) (Chollet et al., 2015). Furthermore, compounds **76a** and **77b** showed medium activities against tuberculosis with MIC values of 10.3 and 21.3 μM, respectively. However, the MIC value of **77c (**1.4 μM) led to further testing against IC2 clinical isolate, which is well resistant toward first- and second-line tuberculosis drugs. According to *in vitro* data, compound **77a** showed less inhibition than GEQ, and the findings from the docking studies also suggest weaker interaction than GEQ.

**SCHEME 18:**
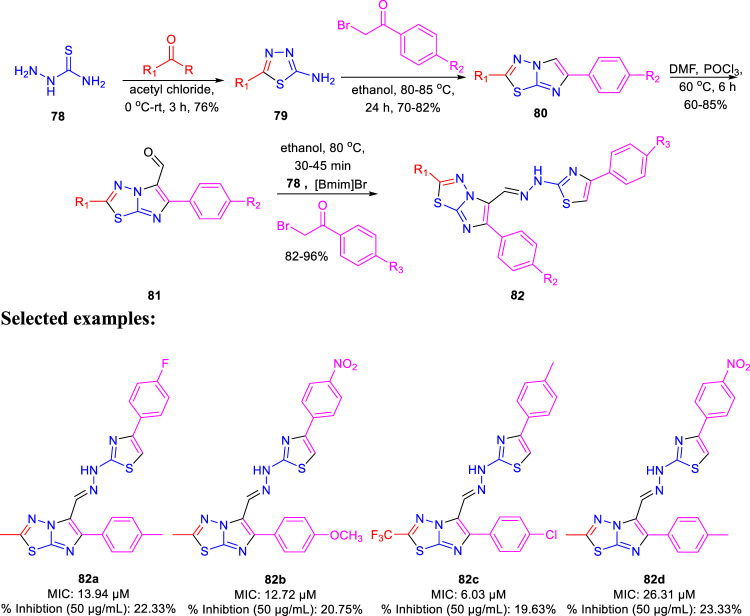
One-pot synthesis of 1-((6-phenylimidazo [2,1-b][1,3,4]thiadiazol-5-yl)methylene)-2-(4-phenylthiazol-2-yl)hydrazine derivatives.

**SCHEME 19:**
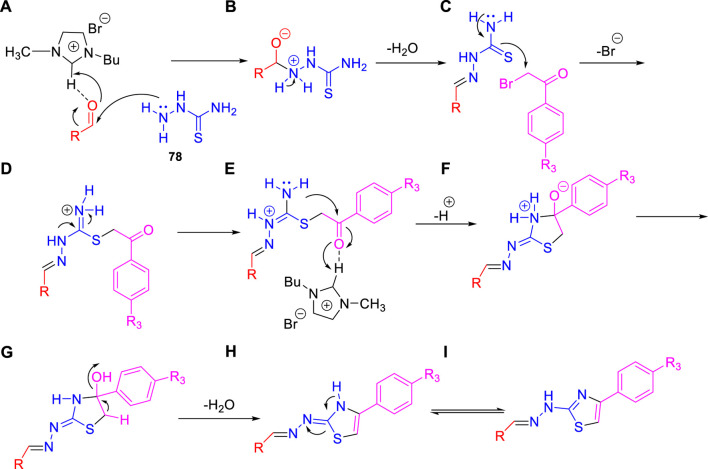
Plausible mechanism for the formation of 1-((6-phenylimidazo [2,1-b][1,3,4]thiadiazol-5-yl)methylene)-2-(4-phenylthiazol-2-yl)hydrazine derivatives.

**FIGURE 8:**
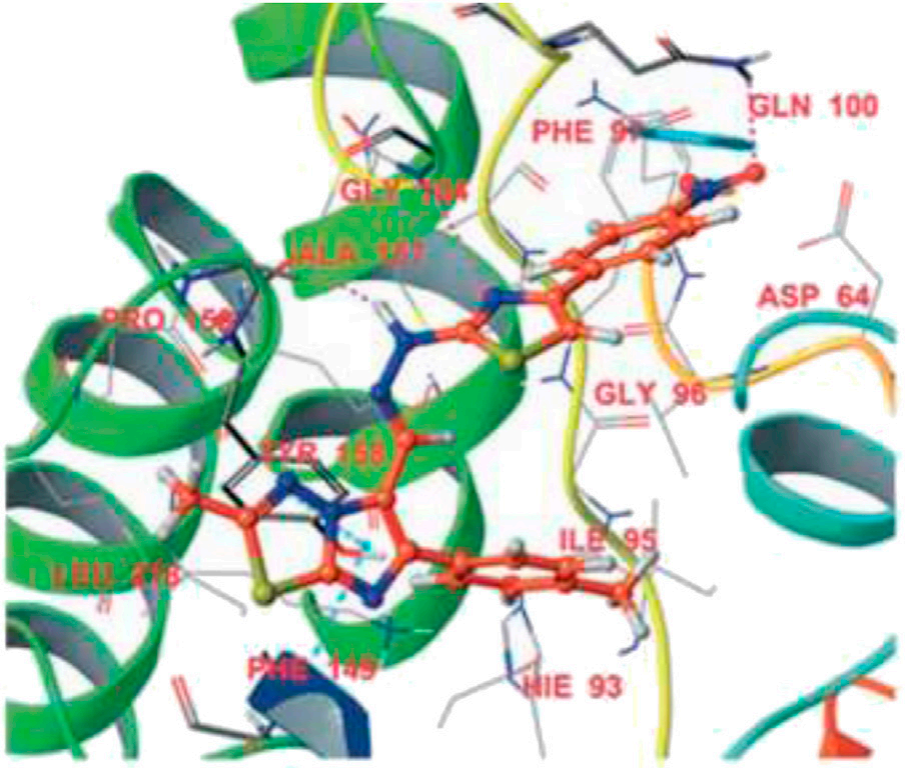
Binding mode of **82d** with InhA. Reproduced from Ramprasad et al., 2016, with permission from Royal Society of Chemistry, Copyright 2016.

Dalimba and co-workers in 2016 reported the one-pot synthesis of thiazole–imidazo [2,1-b] [1,3,4]thiadiazole hybrids and investigated their inhibitory action toward tuberculosis (Ramprasad et al., 2016). Compound **79** is the key intermediate, which was synthesized by the reaction of thiosemicarbazone **78** with ketone in the presence of acetyl chloride. In the presence of ethanol, compound **79** was treated with substituted phenacyl bromide at 80–85°C for 24 h to produce compound **80**, which underwent Vilsmeier–Haack formylation to achieve intermediate **81**. In the last step, treatment of intermediate **81** with substituted phenacyl bromide and thiosemicarbazide **78** in the presence of [Bmim]Br–ethanol mixture furnished different analogs of the final product **82** (Scheme 18) (Alegaon et al., 2012; Ramprasad et al., 2015). A plausible mechanism for the synthesis of 1-((6-phenylimidazo [2,1-b][1,3,4]thiadiazol-5-yl)methylene)-2-(4-phenylthiazol-2-yl)hydrazine derivatives **82** is depicted in Scheme 19. The synthesized moieties were screened against H37Rv strain of *M. tb* using the agar dilution process to determine the antimicrobial activity. Among all of the compounds, **82c** is the most active compound with an MIC value of 6.03 μM, which is better than some of the antitubercular drugs such as ethambutol and ciprofloxacin. However, compounds **82a** and **82b** exhibited moderate activity with MIC values 13.94 and 12.72 μM, respectively. It is interesting to note that all trifluoromethyl derivatives have lower MIC values than the corresponding methyl analogs. Furthermore, promising molecules were docked inside (Figure 8) the active site of the InhA enzyme, and the docking score was observed to be −8.89 kcalmol^−1^, with hydrogen bond interactions along with π–π stacking. The *in vitro* cytotoxicity of the synthesized compounds was examined against NIH/3t3 mouse embryonic fibroblast cell lines using the MTT assay. This study revealed that none of the compounds with significant activity were toxic toward normal cells (Gundersen et al., 2002).

**SCHEME 20:**
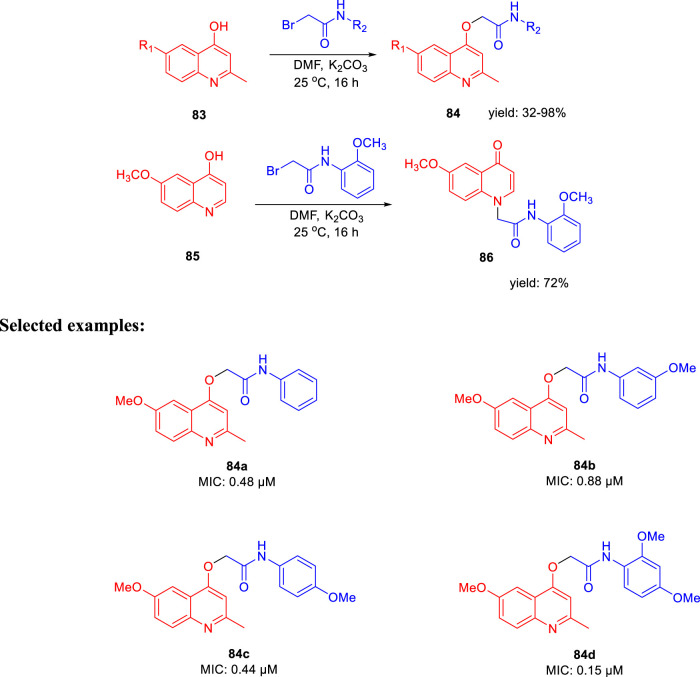
Synthesis of 2-(quinolin-4-yloxy)acetamide derivatives *via* O-alkylation reactions.

Subsequently, Machado and group reported the activity of 2-(quinolin-4-yloxy) acetamides toward drug-resistant and drug-susceptible strains of *M. tb* (Pissinate et al., 2016). As depicted in Scheme 20, compound **84** was synthesized by the *O*-alkylation reactions of compound **83** with 2-bromo-*N*-arylacetamides using K_2_CO_3_ as base in DMF solution at 25°C for 16 h. Moreover, *N-*alkylation of compound **85** with bromo acetamides under the same reaction conditions led to the formation of compound **86** with high chemoselectivity. The whole-cell assay test was performed with the synthesized molecules to evaluate the activities toward H37Rv strain of *M. tb* using isoniazid as standard drug. The result showed that compound **84d** has an exceptional activity with the MIC value as low as 0.15 μM, which is better than those of most first-line antitubercular drugs. Similarly, compounds **84a**, **84b**, and **84c** also have significant activity with 0.48, 0.88, and 0.44 μM MIC values, respectively. All of the synthesized acetamide derivatives showed potency toward drug resistant clinical strain with same activity in infected macrophages. The potential compounds were subjected to evaluation of cardiac toxicity in zebrafish (Selderslaghs et al., 2009) and found to be safe at 1 and 5 μM of the embryos.

**SCHEME 21:**
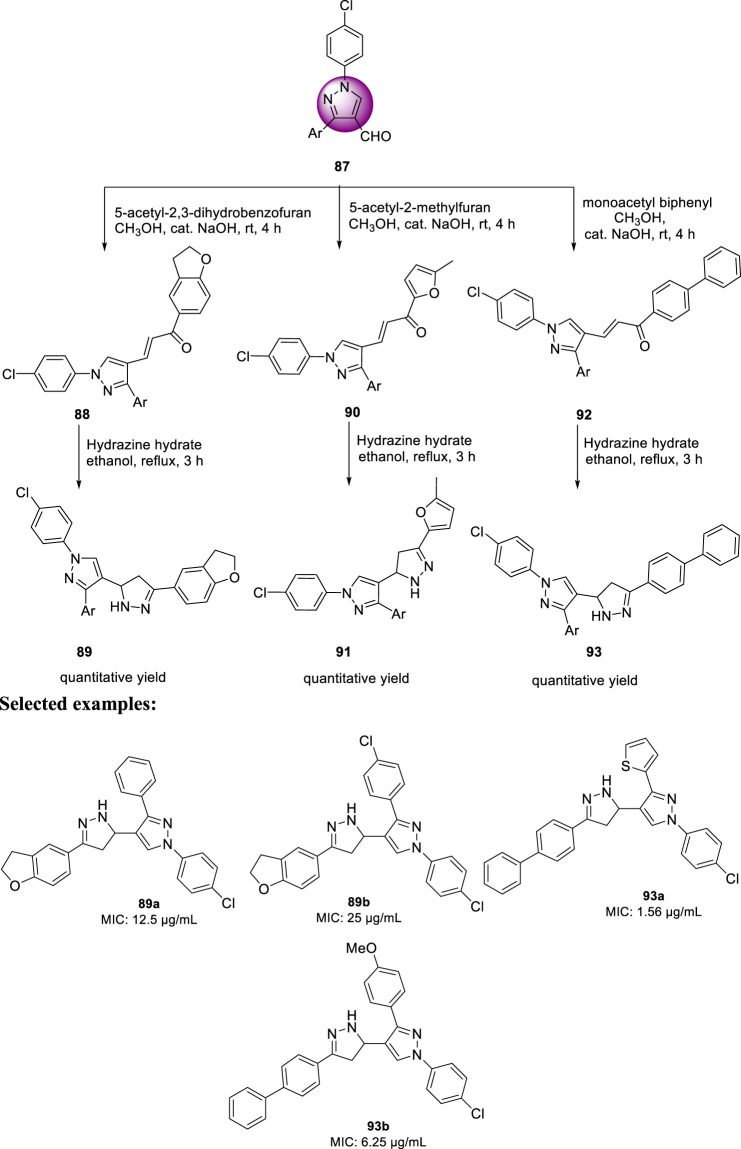
Multistep synthesis of pyrazole-containing pyrazoline derivatives.

In the same year, Isloor *et al.* reported the synthesis of 1′-(4-chlorophenyl) pyrazole bearing 3,5-disubstituted pyrazoline analogs and investigated their antitubercular activities (Harikrishna et al., 2016). Compound **87** is a basic pyrazole skeleton, which can be easily achieved by the Vilsmeier–Haack reaction, leading to excellent yields (Harikrishna et al., 2015). As presented in Scheme 21, pyrazole **87** was treated with 5-acetyl-2,3-dihydrobenzofuran using NaOH as base in methanolic solution at room temperature for 3 h to furnish compound **88**, which upon further treatment with hydrazine hydrate for an additional 3 h in ethanolic solvent afforded the final product **89**. Similarly, compounds **90** and **92** were formed when pyrazole **87** was treated with 5-acetyl-2-methylfuran and monoacetyl biphenyl, respectively, under the same reaction conditions. The synthesized compounds demonstrated a range of MIC values from 50 to 1.56 μg/ml toward H37Rv strain of *M. tb*. Compound **93a** exhibited an MIC value of 1.56 μg/ml, which is better than that of the first-line antitubercular drug streptomycin, whereas compound **93b** has the same MIC value as that of streptomycin, that is, 6.25 μg/ml. Subsequently, compounds **89a** and **89b** exhibited moderate activity with 12.5 and 25 μg/ml MIC values, respectively. An *in vitro* cytotoxicity study was performed with the potential molecules using HeLa cells. After primary screening of the toxicity studies, compounds **83a** and **93b** appeared to be the best potent molecule with minimum cytotoxicity.

**SCHEME 22:**
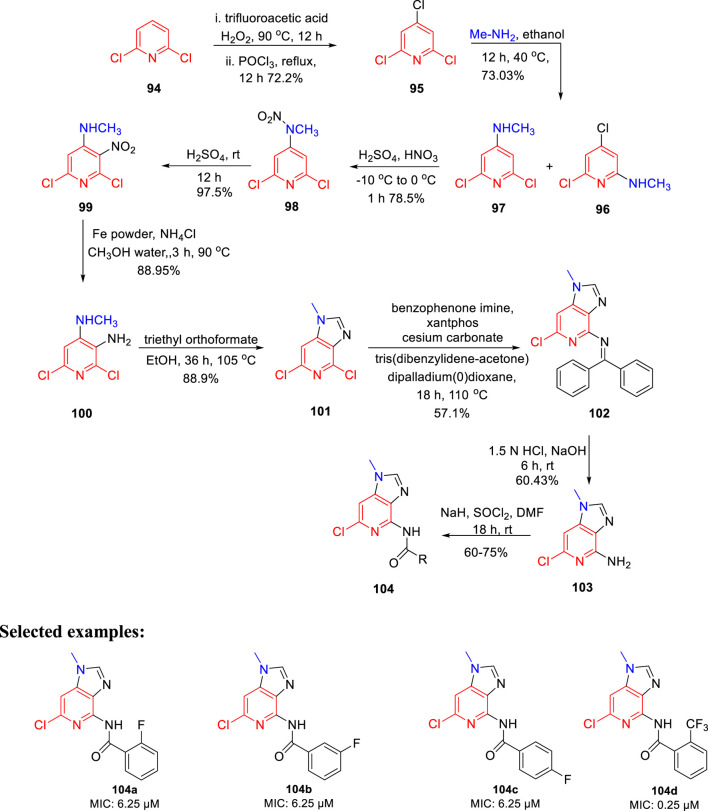
Multistep synthesis of imidazo[4,5-c-]pyridine derivatives.

Prashanth *et al.* synthesized imidazo[4,5-c]pyridine derivatives and evaluated their antimicrobial activity (Madaiah et al., 2016). The synthesis of the desired compound involved a series of reactions initiating from the chlorination of 2,6- dichloropyridine **94** in the presence of trifluoroacetic acid, H_2_O_2_ and POCl_3._ In the next step, treatment of compound **95** with methylamine resulted in the mixture of isomers **96** and **97**. The nitration of compound **97** was performed to afford compound **98**, which upon reaction with sulfuric acid for 12 h at room temperature formed **99**. In the next step, compound **100** was obtained by the reduction of compound **99** using iron powder and ammonium chloride. The imidazole ring of compound **101** was formed by reacting triethyl orthoformate and compound **100** in refluxing ethanol. Furthermore, Buchwald coupling was carried out to achieve the C–N bond formation leading to the synthesis of intermediate **102**. Finally, the amine group in compound **102** was reacted with SOCl_2_ in the presence of sodium hydride to afford the final compound **104** (Scheme 22). All derivatives were screened for their antitubercular activity toward H37Rv strain of *M. tb* by the agar dilution method. Compounds **104a**, **104b**, and **104c** with an MIC value of 6.25 μM expressed more potency than popular anti-TB drug ethambutol (MIC: 7.64 μM). However, compound **104d** has an 0.25 μM MIC value, which is more dominant than isoniazid (MIC: 0.36 μM). The findings suggest that imidazopyridine derivatives might be a promising lead contender against tuberculosis that merit further investigation.

**SCHEME 23:**
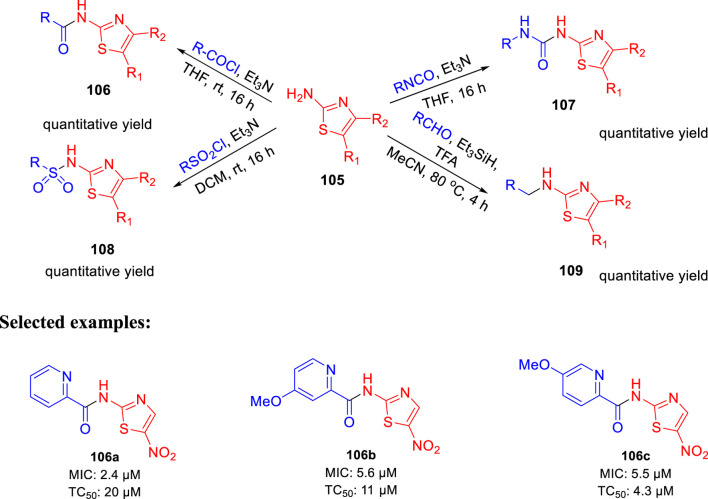
Synthetic route to nitazoxanide derivatives *via* amide bond formation.

In 2017, Parish *et al.* evaluated the *in vitro* activities of unique nitazoxanide (NTZ) derivatives toward *Mycobacterium tuberculosis* with their structure–activity relationship (SAR) (Odingo et al., 2017). In this work, compound **105** was used to regulate the systematic antitubercular SAR study. A total of 56 NTZ (Dubreuil et al., 1996; White, 2004) derivatives were prepared via different pathways using amide bond coupling. As depicted in Scheme 23, treatment of activated acids with aminothiazole **105** afforded compound **106** via amide bond formation. Similarly, reaction of isocyanate with aminothiazole **105** produced compound **107** in the presence of base and THF. On other hand, sulfonyl chloride was used to form sulfonamide derivative **108**. Compound **105** undergoes reductive alkylation reaction in the presence of aldehyde and trifluoroacetic acid to form compound **109**. Minimum inhibitory concentration of compound **106a** was 2.4 μM, whereas compounds **106b** and **106c** have values of around 5.5–5.6 μM toward H37Rv strain of *M. tb*. Toxic concentration (TC_50_) values of the synthesized molecules suggested no significant toxicity.

**SCHEME 24:**
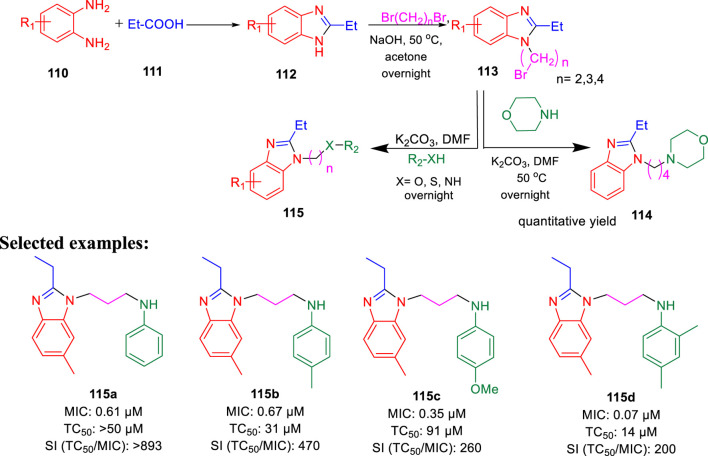
Synthesis of cycloalkyl benzimidazoles *via* a condensation reaction.

Furthermore, the same group accomplished the improved synthesis of phenoxyalkylbenzimidazoles and investigated their potential as the tuberculosis inhibitor to target QcrB (Chandrasekera et al., 2017). Several alkyl derivatives of benzimidazoles were synthesized, as depicted in Scheme 24. Benzimidazole intermediate **112** was formed by the condensation of 1,2- diaminobenzene derivatives **110** with propionic acid **111**. In the next step, the alkylation of intermediate **112** in the presence of dibromoalkane formed *N*-(bromoalkyl)-benzimidazole **113**, which further reacted with anilines, phenols, and thiophenols to form the corresponding benzimidazole alkylamines, alkylethers, and alkylthioethers, respectively. From the SAR study, it was observed that the substitution with 4-methyl did not affect the activity, whereas the substitution with 4-methoxy reduced the activity by four times. Four derivatives **115a**, **115b**, **115c**, and **115d** have selectivity index greater than 200 with MIC values of 0.061, 0.067, 0.35, and 0.070 μM, respectively, toward H37Rv-LP. The cytotoxicity result against the kidney cell line of African green monkey demonstrated that addition of one methyl group in compound **115b** and two methyl groups in **115d** caused a higher increase in toxicity than **115a**. Several synthesized derivatives have better activity inside macrophages in comparison with liquid culture (Chandrasekera et al., 2015).

**FIGURE 9:**
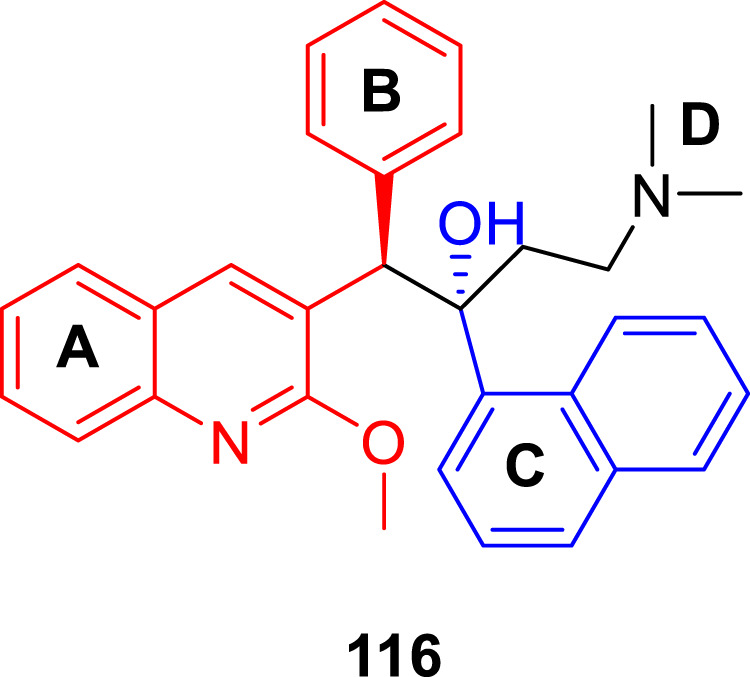
SAR study of bedaquinoline.

Denny *et al.* in the subsequent year synthesized 6-cyano derivatives of bedaquiline as a safe inhibitor of tuberculosis (Tong et al., 2017). The SAR study of almost 200 derivatives was performed to check the significance of four different regions **A**, **B**, **C**, and **D** for antitubercular activity toward the *M. smegmatis* strain (Figure 9) (Guillemont et al., 2011). The IC_90_ of the six-substituted compounds were within a two-fold range in comparison with the lead compound. Most of the compounds were assessed as *RS* and *SR* diastereomers, whereas few molecules were formed as pure *R* and *S* enantiomers.

**SCHEME 25:**
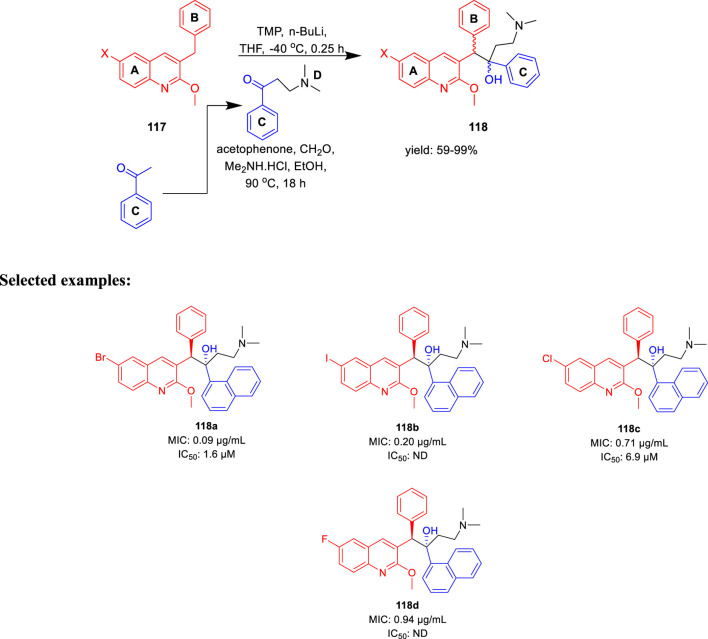
Synthetic route to diarylquinoline derivatives *via* a condensation reaction.

The major diarylquinoline compounds were synthesized by condensation of the proper A/B and C/D units. The C/D unit was achieved using relevant acetophenones in a one-step Mannich reaction (Scheme 25). Compounds **118a**, **118b**, **118c**, and **118d** demonstrated extraordinary MIC values of 0.09, 0.20, 0.71, 0.94 μg/ml, respectively, determined by MABA assay.

**SCHEME 26:**
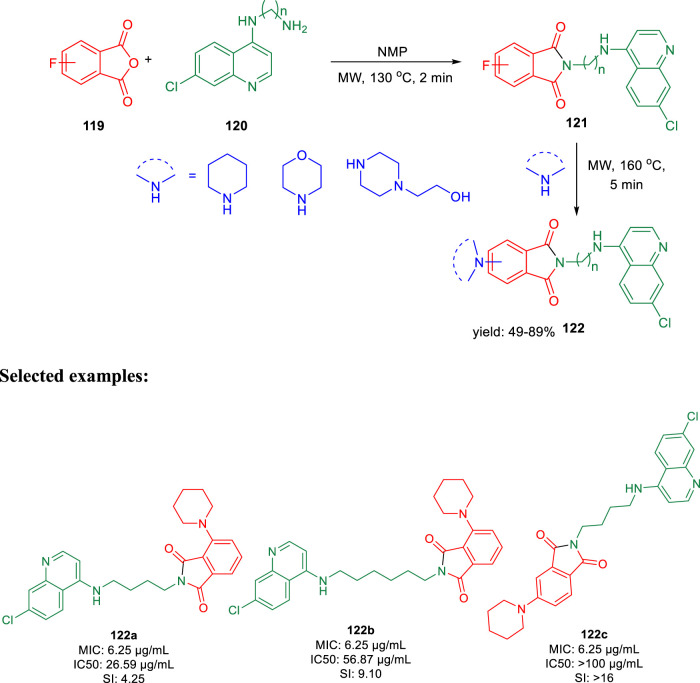
Microwave-assisted synthesis of isoindoline-1,3-dione-4-aminoquinolines (substitution of C-4/C-5 secondary amine).

**SCHEME 27:**
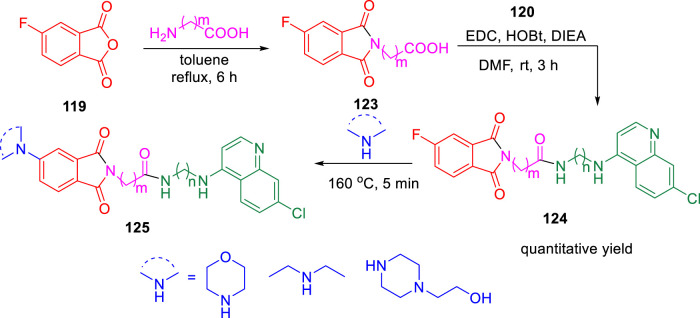
Synthetic route of C-5-substituted isoindoline-1,3-dione connected with 4-aminoquinolines *via* an amide spacer.

In 2019, Kumar and co-authors synthesized substitutedisoindoline-1,3-dione-4-aminoquinolines and evaluated their antimycobacterial properties along with cytotoxicity (Rani et al., 2019). Stepwise microwave-promoted synthesis was performed under optimized conditions to achieve the derivatives of aminoquinolines. Fluoro-phthalic anhydride **119** was treated with 4-aminoquinoline-diamines **120** in the presence of *N*-methylpyrrolidin-2-one to form compound **121**. The addition of secondary amine to compound **121** under microwave irradiation achieved the targeted moiety **122** (Scheme 26). Subsequently, *N*-(7- chloroquinolin- 4- yl)diamine and compound **123** undergo amide coupling with the help of EDC-HOBt to afford molecule **124** at room temperature. Furthermore, the addition of different amines to compound **124** led to the formation of desired C-5-substituted isoindoline-1,2-dione connected with 4-aminoquinolines **125** through an amide spacer (Scheme 27).

**FIGURE 10:**
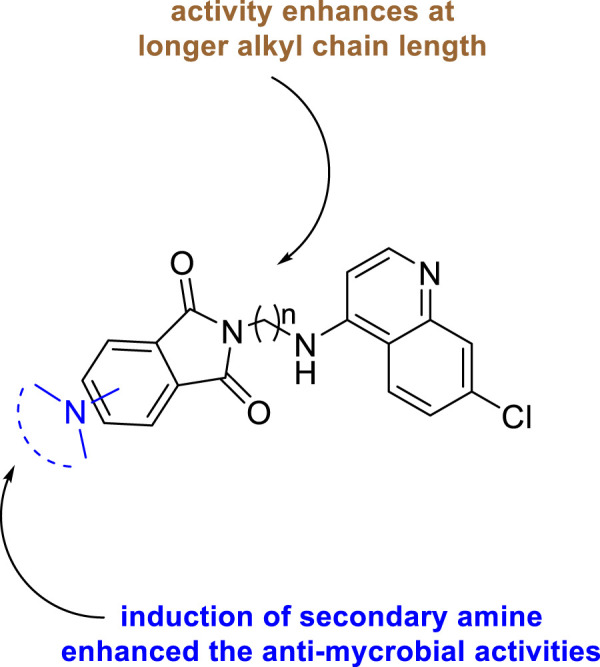
SAR study of isoindoline-1,3-dione-4-aminoquinolines.

Antimicrobial properties of the synthesized compounds were assayed against mc (b)6230 strain of *M. tb*. To analyze the structure–activity relationship, the isoindoline-1,3-dione secondary amine functionality at C4/C5 location and the distance between two pharmacophores were carefully changed. It was observed that the activity was dependent on the type of the linker present between two pharmacophores (Figure 10). However, increase in the alkyl chain length (n = 4, n = 6) and the induction of the morpholine ring resulted in the improvement of activity indicating a 6.25 μM MIC value of compounds **122a**, **122b**, and **122c** with lesser cytotoxicity.

**FIGURE 11:**
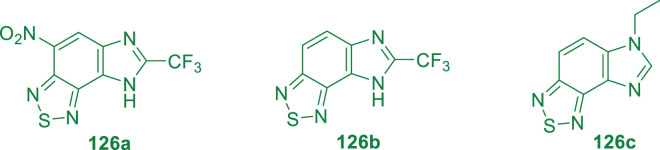
Examples of imidazobenzothiazole.

Parish and his group in 2019 reported the membrane potential disruption of *M. tb* by imidazobenzathiazole analogs (Figure 11) (Smith et al., 2019). Mensuration of membrane potential toward human liver cells in HepG2 was performed by the conventional method where 50,000 cells were plated per well in 96-well plates. However, minimum bactericidal concentration (MBC) was assessed at different pH values, that is, 4.5, 5.6, and 6.8, by fluorescence at different wavelengths.

**FIGURE 12:**
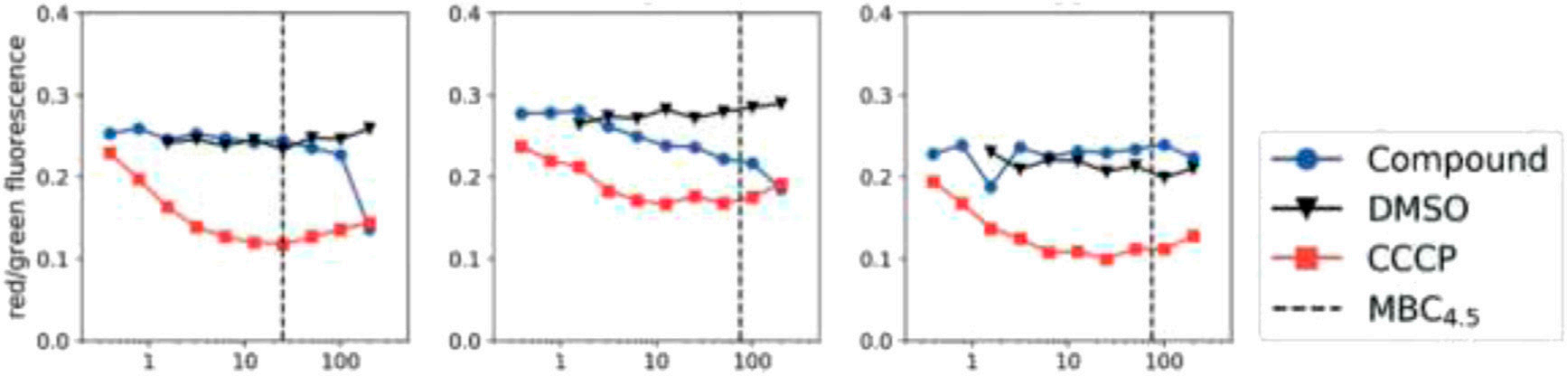
*M. tb* membrane potential disruption at pH 6.8 by benzothiadiazole analogs. Reproduced from Smith et al., 2019, with permission from Royal Society of Chemistry, Copyright 2019.

*M.tb* membrane potential disruption was measured at neutral pH by benzothiazole analogs (Figure 12). The results did not suggest any correlation between the HepG2 IC_50_ value and membrane potential disruption (Huang, 2002). A slight increase in activity was noticed at pH 5.6 for the disruption of *M. tb* membrane potential with higher degree of separation. The perspective of benzothiazole analogs appeared favorable, ruling out the membrane potential disruption for both cytotoxicity and antitubercular activity.

**SCHEME 28:**
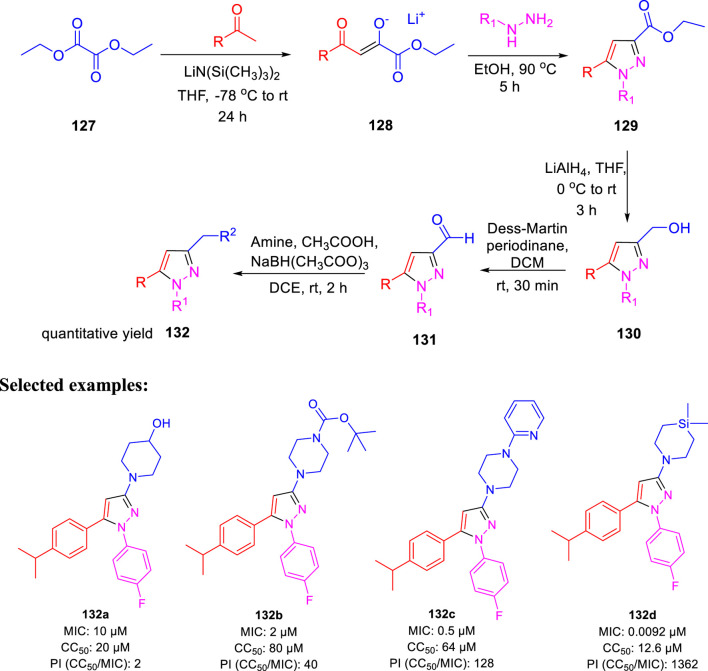
Multistep synthesis of pyrazole bearing derivatives.

In the following year, Poce *et al.* described the effect of pyrazole-containing moieties on *M. tb* by the inhibition of mycobacterium membrane protein large 3, that is, MmpL3 (Poce et al., 2019). The treatment of diethyloxalate **127** with ketones in accordance with lithium bis(trimethylsilyl)-amide formed lithium salt **128**, which further underwent cyclization with relevant hydrazines to produce desired pyrazoles **129**. Pyrazole-3-carbaldehyde **131** was achieved by the conversion of ethyl esters **129** in two steps, as depicted in Scheme 28. Afterward, the reductive amination of **131** with the help of NaBH(CH_3_COO)_3_ in the presence of suitable amine resulted in the formation of pyrazole derivatives **132**. The SAR study was accomplished on a sequence of 1,3,5-trisusbstituted pyrazoles only to find out the significant effect of cyclic amine at the 3-position. Compound **132d**, which has a silicon atom, resulted in an increase of the activity (MIC: 0.00925 μM). Moreover, the genome sequencing results stipulated MmpL3 as a feasible target, confirming the high potential of MmpL3 inhibitors for development in tuberculosis drug discovery (La Rosa et al., 2012).

**SCHEME 29:**
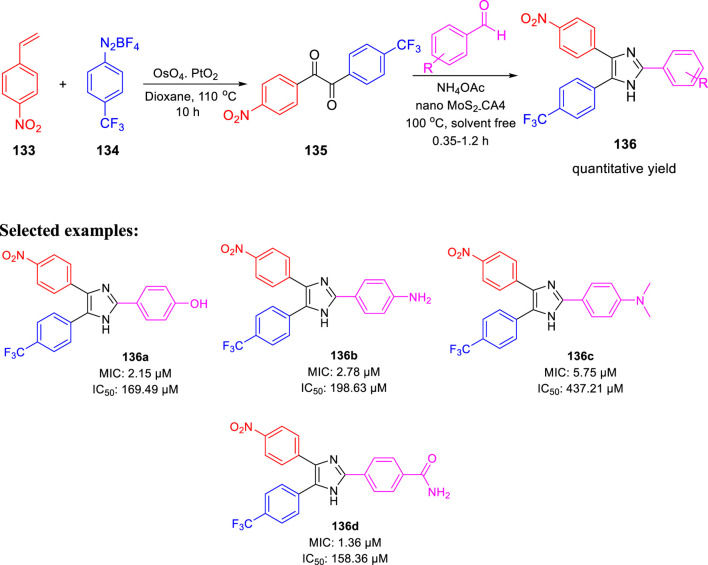
One-pot synthesis of 2,4,5-trisubstituted imidazoles.

In 2020, Prasanth and group accomplished the synthesis of 2,4,5- trisubstituted imidazole derivatives by one-pot methodology employing 4-nitrostyrene and 4-(trifluoromethyl) benzene diazonium salt in the presence of PtO_2_ and OsO_4_ as catalyst (Scheme 29) (Raghu et al., 2020). The pharmacological study indicated significant *in vitro* antitubercular property of compounds **136a**, **136b**, **136c**, and **136d**, revealing MIC values of 2.15, 2.78, 5.75, and 1.36 μM, respectively, toward H37Rv strain of *M. tb*. Very less cytotoxicity was observed with a range of 151.18–437.21 μM IC_50_ values for Vero cells.

**FIGURE 13:**
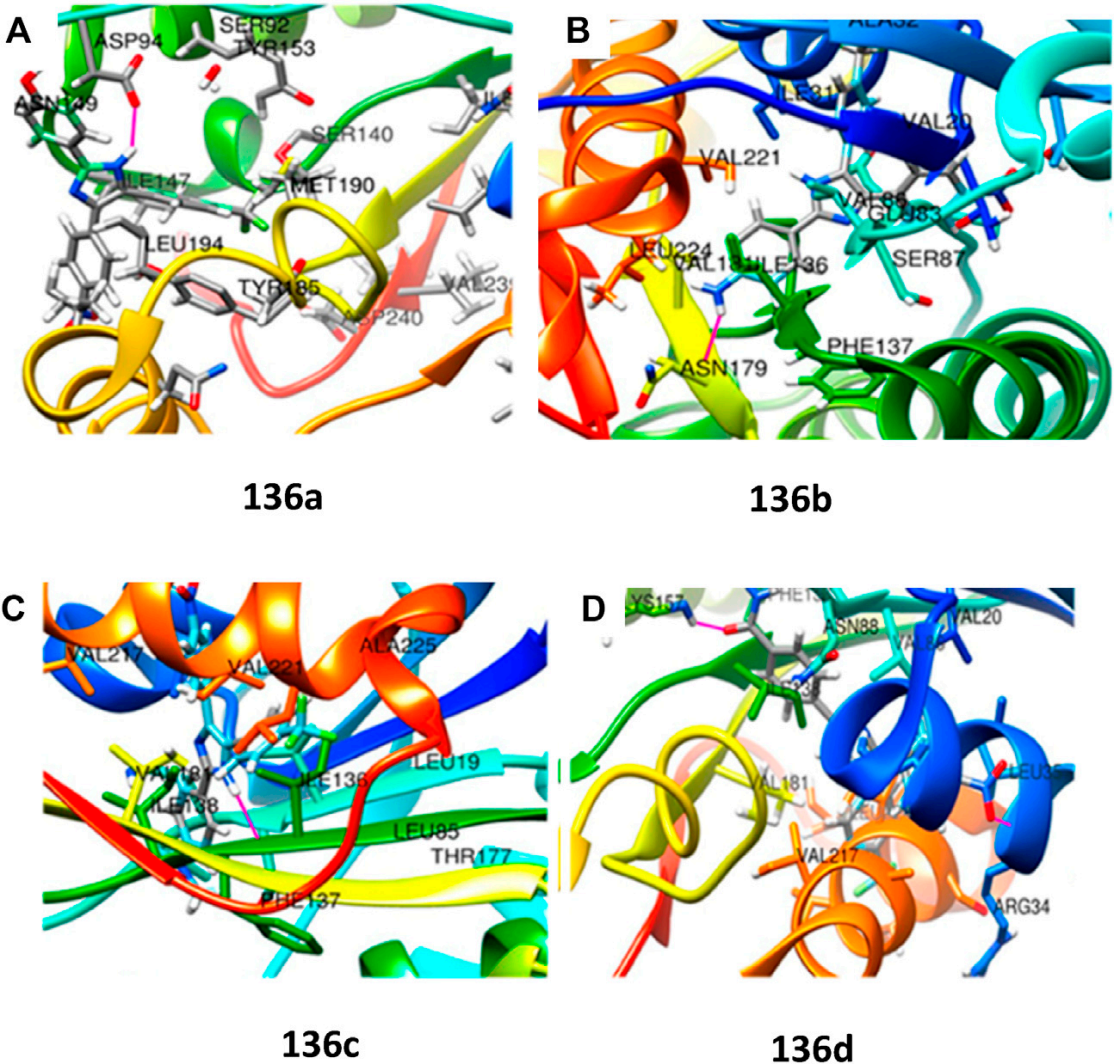
**(A)** Molecular docking of **136a** with MabA protein. **(B)** Molecular docking of **136b** with MabA protein. **(C)** Molecular docking of **136c** with MabA protein. **(D)** Molecular docking of **136d** with MabA protein. Reproduced from Raghu et al., 2020, with permission from American Chemical Society, Copyright 2020.

Moreover, MabA being the key enzyme in biosynthesis of mycolic acid, which is the substantial cell envelop of *M. tb*’s long-chain fatty acid, was selected as an operating site for the docking study. The anticipated binding free energies in kcal/mol were used to determine the molecular docking scores, as presented in Figure 13. The docking scores of **136a**, **136b**, **136c**, and **136d** with 1UZN were 8.5, 8.4, 8.1, and 8.9 kcal/mol, respectively.

**SCHEME 30:**
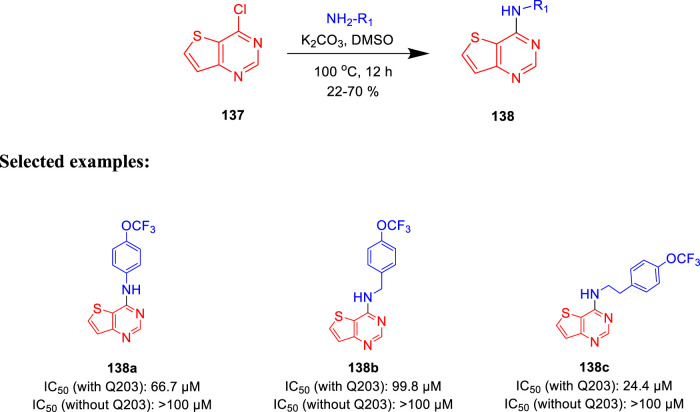
One step synthesis of thieno [3,2-*d*]pyrimidin-4-amines.

Moraski *et al.* generated the structure-guided thieno[3,2-d]pyrimidine-4-amine and investigated its potency as bd oxidase inhibitors of *M*. *tb* (Hopfner et al., 2021). Nucleophilic aromatic substitution (S_N_Ar) reaction was implemented with 4-chlorothieno [3,2-d] pyridine and amines at 100°C in the presence of base, as depicted in Scheme 30 (Neri et al., 2020). Thirteen synthesized analogs were screened in a whole-cell ATP cyt-bd assay. The test was performed under replicating circumstances with and without the addition of Q203 in the H37Rv-*M.tb* strain and the clinical isolate N0145-*M.tb*. Subsequently, the BCG strain was utilized for the identification of any common cyt-bd inhibitor. The result shows that H37Rv-*M.tb* strain has overexpressed cyt-bd in comparison with the clinical isolate. Weak potency of the synthesized molecules was increased by functionalizing the para position of the Ph group, making compound **138c** the most potent one. This class of synthetically accessible compounds is one of the rare published examples which can successfully inhibit the cyt-bd in mycobacteria.

**SCHEME 31:**
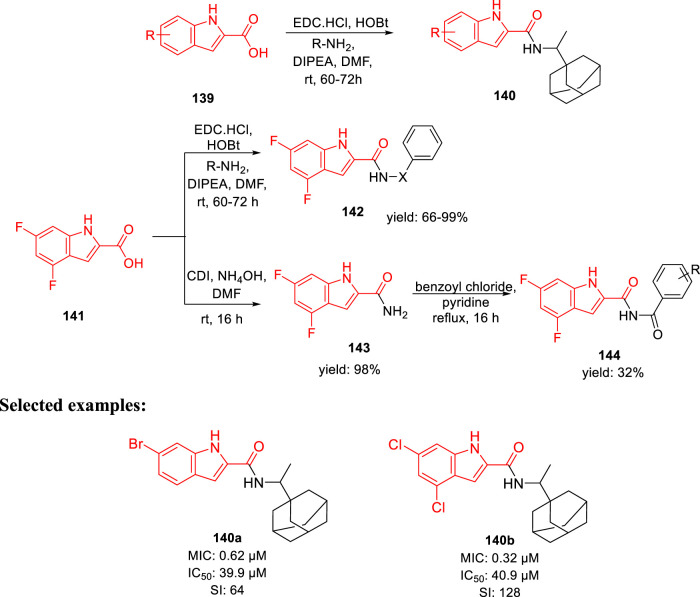
Synthesis of indole-2-carboxamides *via* amide coupling.

Gunosewoyo *et al.* synthesized indole-2-carboxamides as inhibitor of *Mycobacterium tuberculosis* in 2021 (Alsayed et al., 2021). To produce *N*-rimantadine indoleamides **140**, amide coupling of compound **139** was performed in accordance with DIPEA, EDC HCl, and HOBt. Subsequently, compound **142** was synthesized from 4,6- difluoroindole-2-carboxylic acid **141** *via* amide coupling with appropriate amine. Likewise, the reaction of **141** with 1,1′-carbonyldiimidazole resulted in the formation of *N-*acylimidazole intermediate, which upon *in situ* treatment with ammonium hydroxide achieved the corresponding amide **143** *via* a nucleophilic substitution reaction. Finally, targeted moiety **144** was synthesized by the reaction of benzoyl chloride derivatives with compound **143** in pyridine (Scheme 31). Compounds **140a** and **140b** exhibited the MIC values of 0.62 and 0.32 μM, respectively, for H37Rv strain. An increase in efficacy with high lipophilic groups was noted, whereas 5-methoxy derivatives indicated two times reduced activity compared to 4-methoxy derivatives in the SAR study. The high lipophilic character of the preceding series resulted in diffusion *via* the lipid-rich bilayer of *M. tuberculosis* by their possible interaction with MmpL3 to enhance the anti-TB activity.

**FIGURE 14:**
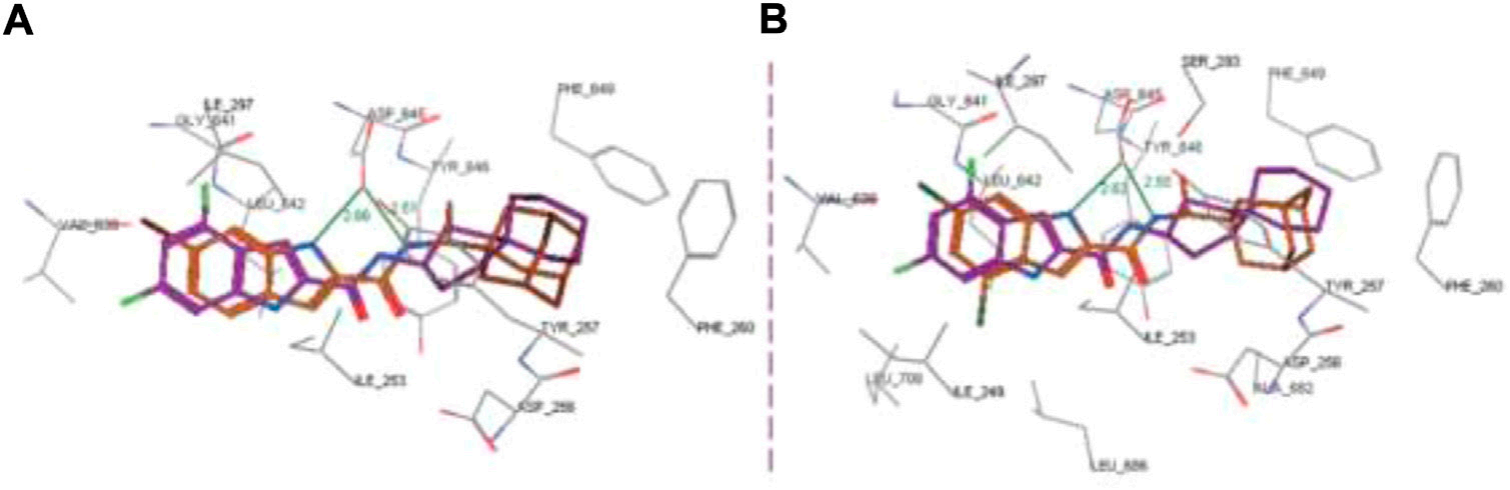
Superposition of top-ranked docking pattern of **140(A)** and **140(B)**. Reproduced from Alsayed et al., 2021, with permission from Royal Society of Chemistry, Copyright 2021.

In the docking study, as depicted in Figure 14, the S3 hydrophobic site was embedded in the indole moiety, whereas the rimantadine nucleus was inserted in the hydrophobic bulky S5 subsite. On the other hand, the amide NH was accommodated in the hydrophilic S4 subsite. The same binding pattern of compounds **140a** and **140b** and the MmpL3 inhibitor **ICA38** stipulated potency through disruption of Asp-Tyr pairs, which is the major player for proton dislocation. These findings suggest that indoleamides can be considered a new class of antitubercular agents.

**SCHEME 32:**
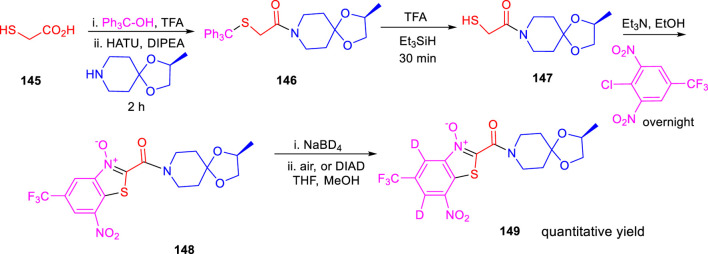
Synthesis of BTO with BTZ043 side chain with reduction reoxidation chemistry.

Miller and his research group reported the formation of hybrid-induced Meisenheimer’s complex reflecting the efficacy as an antituberculosis agent in the subsequent year (Liu et al., 2021). BTO analog **148** resembles BTZ043 due to the incorporation of an identical piperidine acetal unit. It was prepared according to previously reported synthetic protocols, which involved the nucleophilic aromatic substitution reaction as a key step followed by cyclization (Kozikowski et al., 2007). Treatment of NaBD_4_ with compound **148** followed by oxidation generated the deuterium-incorporated starting material (Scheme 32). The intermediates can act as precursors toward the indispensable nitroso moiety, which forms covalent adducts to inhibit DprE1 enzyme. Evaluation of the effects of compounds was assessed by metabolic labeling of H37Rv strain of *M. tb*. The findings suggested the necessity of the incorporation of highly electron deficient substituents to facilitate the molecular recognition by target enzyme (Liu et al., 2019). These compounds have the ability to form the Meisenheimer complex quickly by reacting with hybrids. Interestingly, radiolabeled lipid demonstrated DprE1-related activity during agglomeration of trehalose monomycolates and trehalose dimycolates, and this is due to the scarcity of arabinan chains acting mycolates attachment sites in the mycobacterial cell wall (Landge et al., 2015). The result indicates that further investigation is needed to broaden the scope for the development of potent nitro-substituted antitubercular drugs.

**SCHEME 33:**
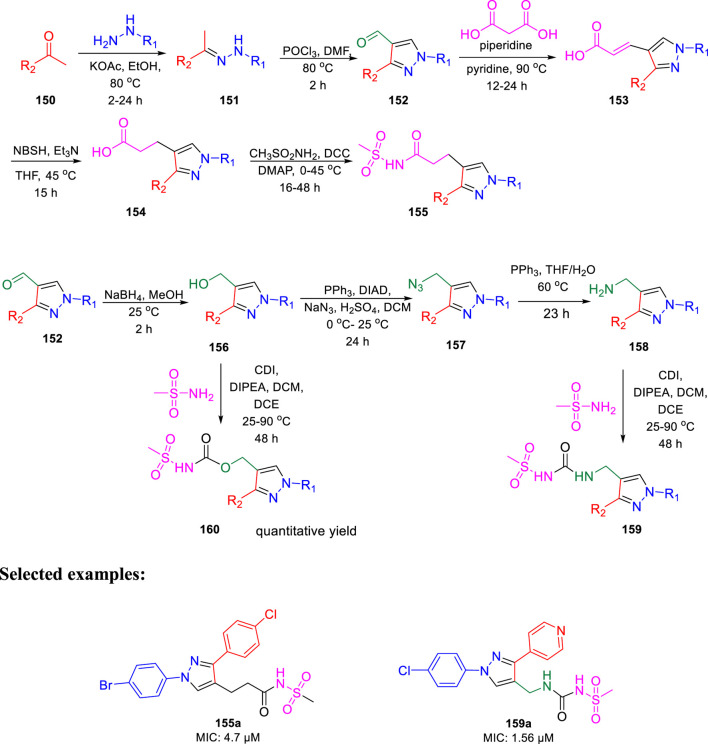
Multistep synthesis of 1,3-diarylpyrazolyl-acylsulfonamides.

In the same year, Chibale *et al.* reported the ability of 1,3-diarylpyrazolyl-acylsulfonamides to target cell wall biosynthesis of *M. tuberculosis* (Khonde et al., 2021). Several targeted compounds have been synthesized *via* a series of reactions, as depicted in Scheme 33. The mode of action of synthesized derivatives was assessed to check the inhibitory actions toward various strains of tuberculosis. PiniB-LUX bioluminescence assay (Naran et al., 2016) revealed disruption of cell wall biosynthesis by modulating the expression of *ini*BAC operon. This was additionally confirmed by the transcriptional profile study, which indicated the upregulation in the genes that are involved in cell wall biosynthesis. *In vivo* studies indicated the moderate inhibition of intracellular replication of tuberculosis in lungs, whereas the *in vitro* studies specified the adequate stability along with significant plasma protein binding. Compounds **155a** and **159a** showed notable MIC values of 4.7 and 1.56 μM, respectively, with considerable cytotoxicity.

**SCHEME 34:**
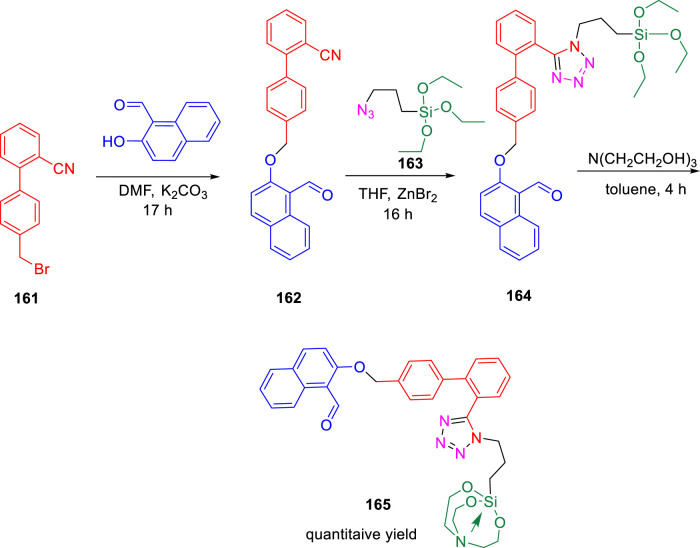
Three-step synthetic pathway to tetrazole-appended ether derived organosilane and organosilatrane.

During that year, Singh *et al.* accomplished the tetrazole coalesced organosilane as enoyl ACP reductase inhibitor for *Mycobacterium tuberculosis* (Singh et al., 2022). Tetrazole conjoined molecules were synthesized *via* three-step process using 3-azidopropyltriethoxysilane **163** in the presence of catalytic ZnBr_2_, as shown in Scheme 34.

**FIGURE 15:**
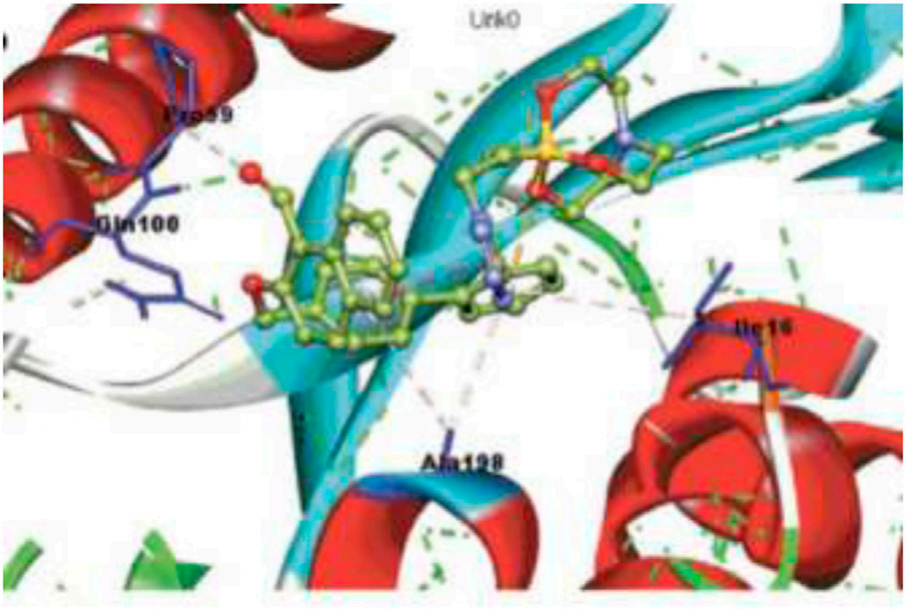
Binding pattern of **165** with InhA. Reproduced from Singh et al., 2022, with permission from Royal Society of Chemistry, Copyright 2022.

The docking study of the molecules indicated the successful binding at the active site of the InhA enzyme with hydrogen bond interactions and pi–pi interactions. The various types of interaction lead to binding energy as good as −7.82 kcal mol^−1^ with a 0.00 RMSD value, as depicted in Figure 15.

**SCHEME 35:**
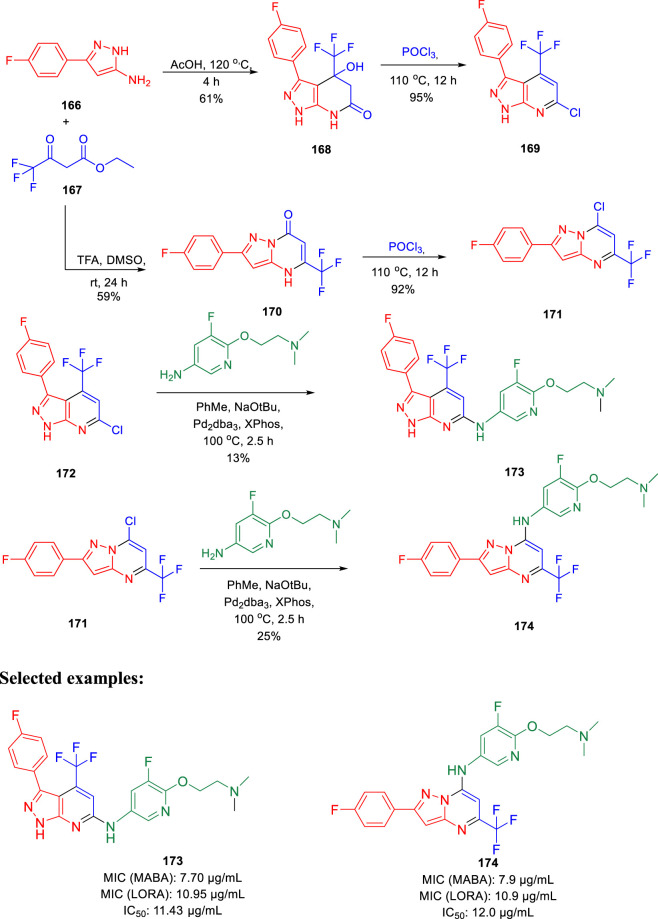
Synthetic path to pyrazolo[3,4-β]pyrimidine and pyrazolo [1,5-α]pyrimidine.

Choi *et al.* synthesized the pyrazolopyrimidines and assessed its potential as ATP synthesis inhibitors of tuberculosis (Choi et al., 2022). Pyrazolo[3,4-β]-pyrimidinone **168** and pyrazolo[1,5-α]-pyrimidinone **170** were transformed to the relevant chlorides **169** and **171**, respectively, in the presence of phosphorus oxychloride at 110°C. The desired products pyrazolo[3,4-β]-pyrimidine **173** and pyrazolo[1,5-α]-pyrimidine **174** were produced by the Buchwald– Hartwig amination reaction, as depicted in Scheme 35 (Tantry et al., 2016). Pyrazolo[3,4-β]-pyrimidine **173** and pyrazolo [1,5-α]-pyrimidines **174** were tested against both replicating aerobic (MABA) and nonreplicating anerobic (LORA) cultures of *Mycobacterium tuberculosis.* The findings suggest that both the analogs demonstrated medium activity with MIC_90_ values of ∼8 μg/ml against MABA and ∼11 μg/ml against LORA bacterial cultures. Bedaquiline, which is a well-known inhibitor of ATP synthase to cure multidrug-resistant tuberculosis, was used as a standard inhibitor in this work. Further mammalian cell toxicity study in the epithelial kidney cells of green monkey displayed that both compounds **173** and **174** have 11–12 μg/ml IC_50_ values. Moderate activity with low cytotoxicity makes them potential candidates for the ATP synthesis inhibitor of *Mycobacterium tuberculosis*.

**SCHEME 36:**
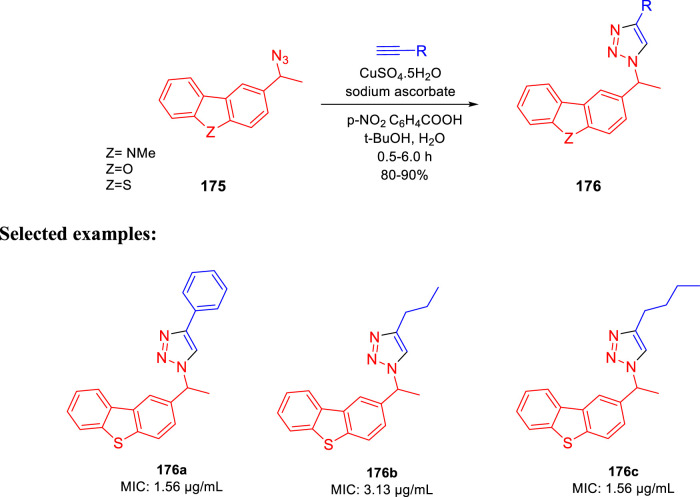
Synthesis of azides *via* click reaction.

Kantevari and co-authors accomplished the synthesis of dibenzo[*b,d*]thiophene, dibenzo[*b,d*]furan, and *N*-methylcarbazole clubbed 1,2,3-triazoles and evaluated their efficacy as the tuberculosis inhibitor (Patpi et al., 2012); 1,3- dipolar cycloaddition reaction between azides **175** and alkynes led to the formation of desired clubbed triazole moieties in the click pathway. Several derivatives of **176** were synthesized by incorporating different heteroatoms as Z groups, that is, S, O, and N-Me, to accomplish the targeted dibenzo[*b,d*]thiophene, dibenzo [*b,d*]furan and *N*-methylcarbazole-clubbed 1,2,3-triazoles (Scheme 36). Both **176a** and **176c** demonstrated significant inhibition with a 1.56 μg/ml MIC value toward H37Rv (ATCC 27294) strain. The cytotoxicity of the dominant compounds was judged using four different cell lines, that is, A549 (adenocarcinomic human lung epithelial cell), DU145 (human prostate cancer), HeLa (human cervical carcinoma epithelial cells), and SK-N-SH (human neuroblastoma) *via* MTT assay to obtain SI values ranging from 55 to 255. Several derivatives had MIC values less than 6.25 μg/ml, which is a value proposed by the global program for the invention of novel antituberculosis drugs as an absolute maximum for evaluating novel *M. tb* therapies.

**SCHEME 37:**
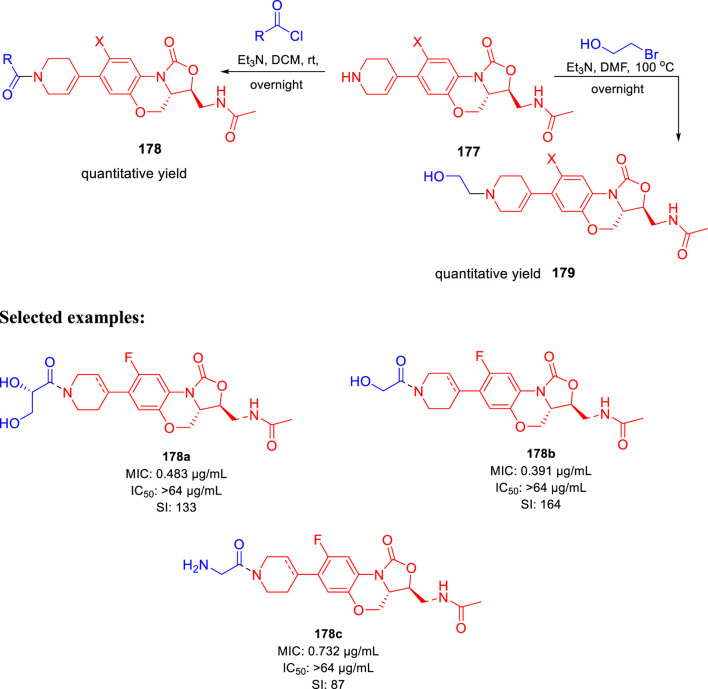
Multistep synthesis of fluorine bearing benzoxazinyl-oxazolidinones.

**FIGURE 16:**
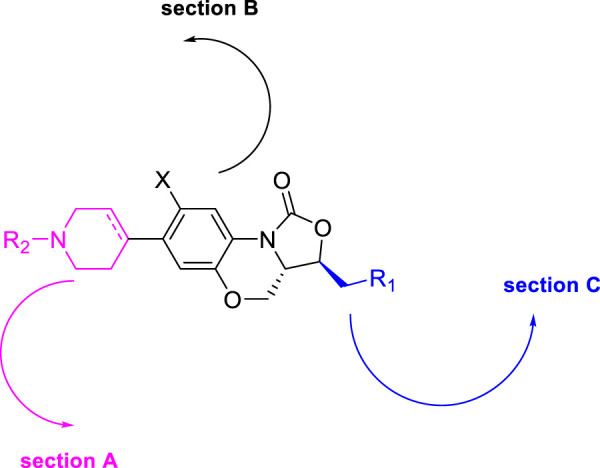
SAR study of the target compound.

Huang *et al.* reported the fluorine incorporated benzoxazinyl-oxazolidinone derivatives to treat multidrug-resistant tuberculosis (Zhao et al., 2017). The addition of cyclopropanecarbonyl chloride to compound **177** in the presence of triethylamine formed compound **178** at room temperature. Similarly, compound **179** was formed by the addition of 2-bromoethanol to compound **177** at an elevated temperature of 100°C using triethylamine as base (Scheme 37). A small flexible hydrophilic group appeared to be favorable for antitubercular action from the SAR study. 2-Hydroxyacetyl and 2-hydroxyethyl groups in A section and the acetylamino group in C section were chosen as optimal fragments. They helped investigate the effect of fluorine on the benzene ring and double bond in the tetrahydropyridine ring, as presented in Figure 16. The influence of these three different sections on the cytotoxicity and potency was thoroughly studied in this work.

Few compounds were chosen to examine the potential anti-DR-TB activity due to their strong potency toward H37Rv strain and outstanding selectivity index values. *In vitro* potency against the 16892 strain was found to be very satisfying in most of the tested compounds. The compound’s effectiveness against the 16802 strain (XDR-TB) was equally encouraging, and it can be now promoted to the next round of clinical trial.

**SCHEME 38:**
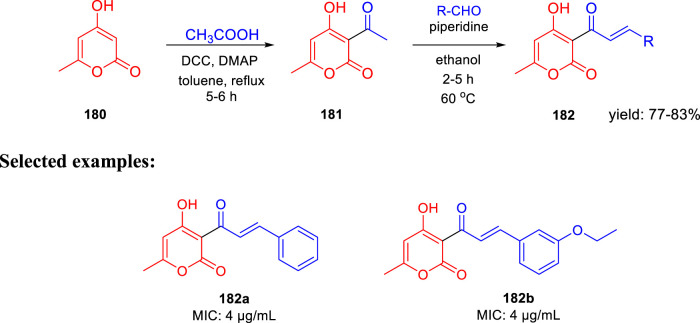
Piperidine catalyzed synthetic path to 3-cinnamoyl-4-hydroxy-6-methyl-2*H*-pyran-2-one anagrams.

In search of a new antituberculosis agent, Yousuf and his team synthesized and established an *in vitro* assessment of substituted 3-cinnamoyl-4-hydroxy-pyran-2-one (CHP) toward *M. tb* (Bhat et al., 2018). As depicted in Scheme 38, 4-hydroxy-6-methyl-pyran-2-one **180** was refluxed with acetic acid using DCC and DMAP in toluene to achieve the 3-acetyl-4-hydroxy-6-methyl-pyran-2-one **181.** Compound **181** was then further reacted with suitable aldehydes in the presence of piperidine to afford the desired CHP **182**. The MIC value of 4 μg/ml was found in two compounds, that is, **182a** and **182b**, indicating excellent antituberculosis activity against *M. tuberculosis*. These MIC values are close to those found in the standard antitubercular drugs EMB, STR, and LVX, suggesting that these two compounds need further investigation. *M. tuberculosis* cell walls are supposed to contain small polyketide molecules, which can regulate permeability. 2-Pyrone polyketides, a diverse class of secondary metabolites that play critical roles in *M. tuberculosis*, may be responsible for this significant antituberculosis activity (Saxena et al., 2003; Gokulan et al., 2013).

**SCHEME 39:**
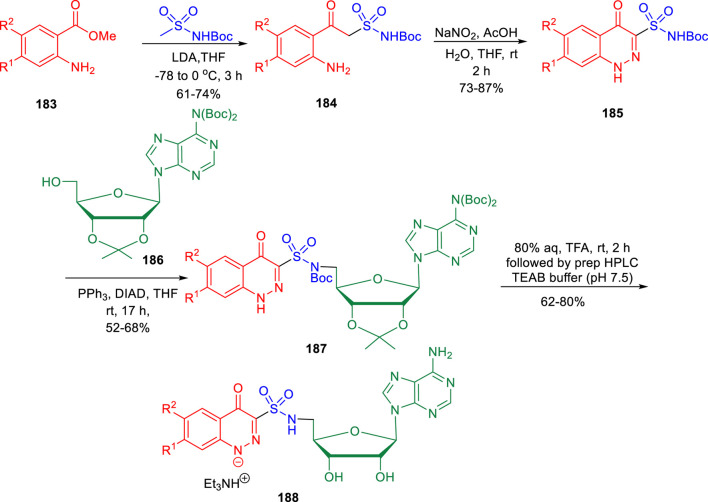
Multistep synthetic route to cinnoline nucleoside.

In the subsequent year, conformationally strained cinnoline nucleosides were reported by Aldrich and his group as tuberculosis siderophore biosynthesis inhibitors (Dawadi et al., 2018). Anthranilic acid methyl ester **183** underwent Claisen-like condensation with the dianion of *N-*Boc methyl sulfonamide to achieve β-ketosulfonamide compound **184**, which upon diazotization in a mixture of AcOH-H_2_O-THF solvent led to the formation of cinnoline-4-one-3-sulfonamide **185**. In the next step, a regioselective Mitsunobu coupling reaction of compound **185** with bis-Boc adenosine **186** led to the formation of **187**. Deprotection of compound **187** by aqueous TFA afforded the desired cinnoline nucleoside **188** (Scheme 39). A [^32^P]PP_i_-ATP exchange assay with salicylic acid and ATP at physiologically relevant supersaturation concentrations was used to evaluate the inhibition of recombinant MbtA by the synthesized compounds (Somu et al., 2006). According to the Morrison equation, the apparent inhibition constant of tight-binding inhibitors was determined by fitting the concentration–response plot to the Morrison equation (appK_i_).

**FIGURE 17:**
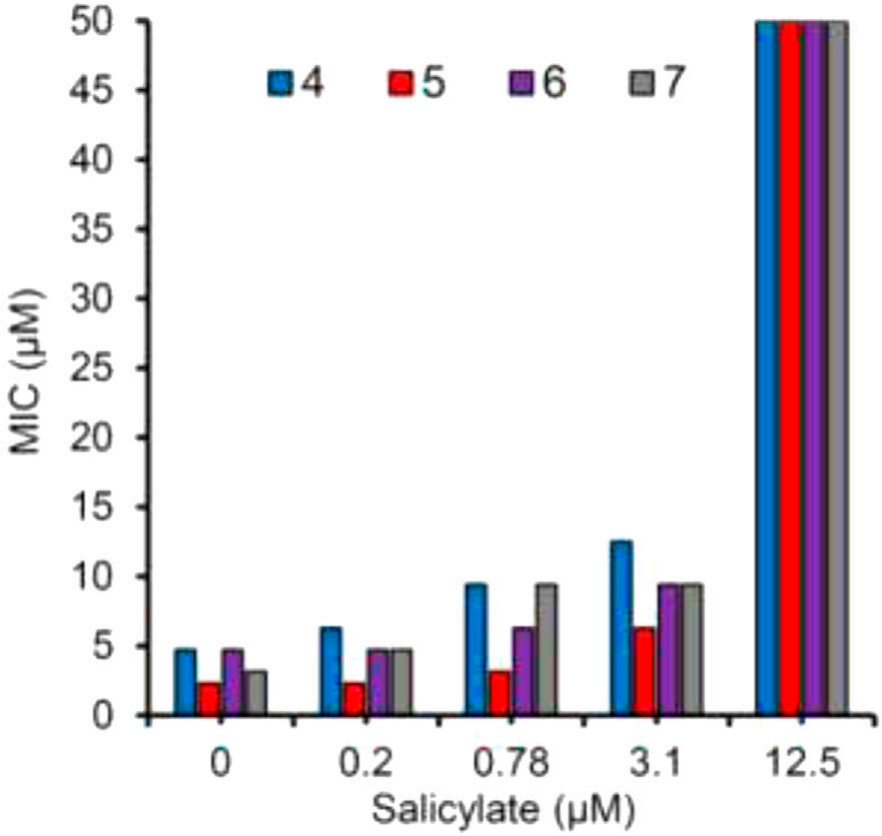
Growth sensitivity of *M. tb* in GAST medium supplemented with salicylate at 0, 0.2, 0.78, 3.1, and 12.5 μM. Reproduced from Dawadi et al., 2018, with permission from American Chemical Society, Copyright 2018.

Glycerol–alanine–salt (GAS) medium was used to test the whole-cell activity of H37Rv strain of *M. tuberculosis*. Biochemical data supported the design strategy by showing that the MICs required to inhibit 99.999% of bacterial growth ranged from 2.3to 4.7 μM, as depicted in Figure 17.

**SCHEME 40:**
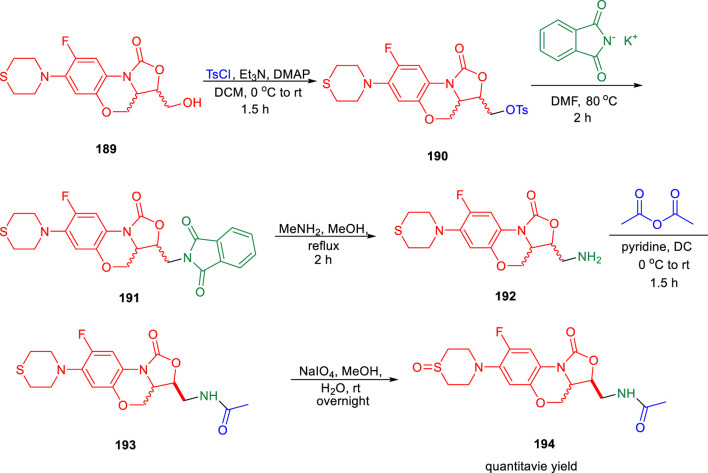
Multistep synthesis of constrained oxazolidinones.

Lu *et al.* reported the conformationally strained oxazolidinone for treating multidrug-resistant tuberculosis with improved safety and efficacy profiles (Zhao et al., 2020). Reaction of compound **189** with tosyl chloride resulted in the formation of intermediate **190**, which further underwent nucleophilic substitution in the presence of potassium phthalimide to obtain moiety **191**. In the next step, compound **191** was transformed to intermediate **192** by the addition of methylamine in methanol. Furthermore, acetylation reaction of intermediate **192** led to the formation of **193**, which was transformed into corresponding sulfoxide **194** by the addition of NaIO_4_ in methanol (Scheme 40). Compound **193** and its sulfoxide metabolite **194** were tested against drug-resistant *M. tuberculosis* strains using sutezolid and linezolid as standard, based on their strong efficacy against H37Rv, modest MPS inhibition, and good microsome stability. The findings indicated that the potency of **193** is much higher than that of **194**, sutezolid, and linezolid. The MIC value of **193** is 4–10-fold higher in the linezolid-resistant, that is, L-R strain than in the H37Rv strain. These findings suggest that compound **193** is likely to bind to the same location as other oxazolidinones. Several *in vitro* ADME experiments were run on compound **193** to learn more about its drug ability. In hepatocytes from several species, compound **193** demonstrated high metabolic stability. The CYP450 enzymes CYP1A2, CYP2D6, CYP2C9, CYP2C19, and CYP3A4 were also examined with compound **193**. The IC_50_ values against all of these CYP450 isoforms were all greater than 45 μM, which indicates that they have a low risk of drug–drug interactions.

**SCHEME 41:**
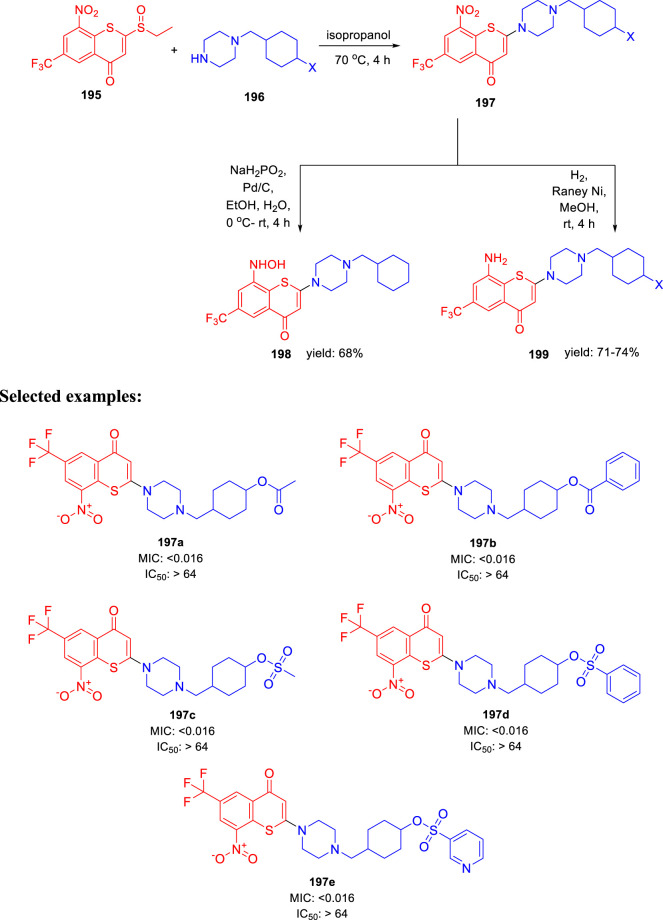
Synthesis of target metabolites *via* reduction.

In 2021, Yu Lu and coworkers accomplished the synthesis of benzothiopyranone derivatives, containing amide and ester groups as promising leads toward tuberculosis (Li et al., 2021). Compound **195** was synthesized according to the previously reported synthetic procedures (Li et al., 2019). Treatment of substituted cyclohexylmethylene piperazine **196** with compound **195** in the presence of isopropanol at 70°C to form compound **197** via an y addition elimination strategy. By reducing the nitro group in compound **197** with the help of Pd/C and NaH_2_PO_2_, hydroxylamine derivative **198** was formed. On the other hand, Raney nickel hydrogenation was performed to transform the nitro group of compound **197** into corresponding amine **199** (Scheme 41). Representative compounds were evaluated against two XDR-TB clinical isolates based on their efficacy against *M. tuberculosis* H37Rv strain. Compounds **197a** and **197b** with an ester motif displayed potent activity against these strains. Furthermore, **197c**, **197d**, and **197e** compounds containing a sulfonate motif showed very significant efficacy against drug-susceptible and drug-resistant tuberculosis. The SAR study indicated that the involvement of small five-membered aromatic heterocycles, that is, furan and thiophene, increased the potency of the molecules.

**FIGURE 18:**
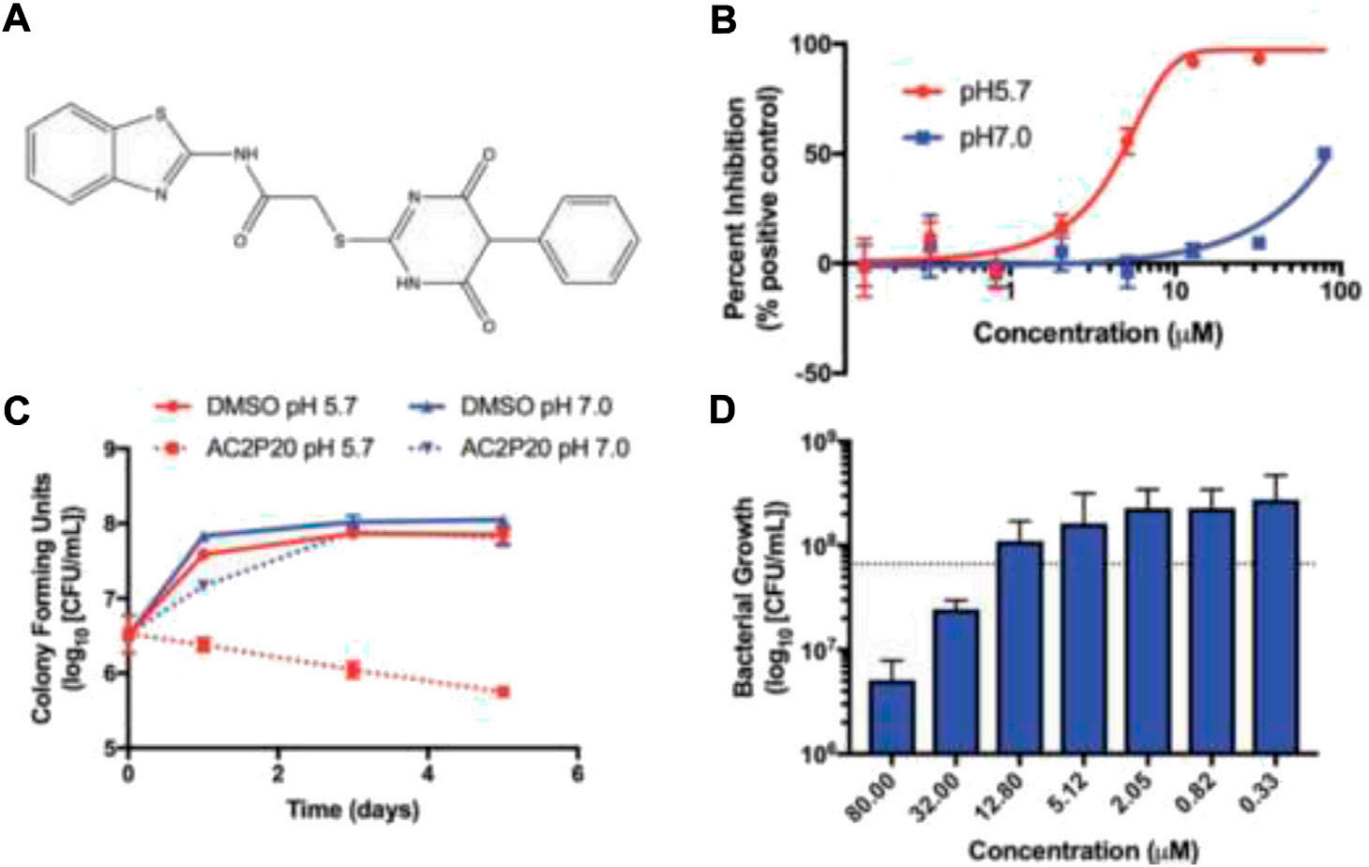
**(A)** Structure of AC2P20, **(B)** dose dependence of *M. tb* growth inhibition with the treatment of AC2P20 at 5.7 and 7.0 pH and exhibition of 4.3 μM EC_50_ after a treatment that lasted for 6 days, **(C)** treatment of 20 μM AC2P20 with *M. tb* for 5 days in a time-dependent way, and **(D)** treatment of AC2P20 with *M. tb* for 7 days at 5.7 pH in a dose-dependent way. Reproduced from Dechow et al., 2021, with permission from Royal Society of Chemistry, Copyright 2021.

Abramovitch *et al.* in 2021 established that AC2P20 (N-1,3-benzothiazol-2-yl-2-[(4,6- dioxo-5-phenyl-1,4,5,6-tetrahydropyrimidin-2-yl)thio] is selective in killing *Mycobacterium tuberculosis* at acidic pH through the depletion of free thiols (Dechow et al., 2021). Two *M. tuberculosis* strains, Erdman and CDC1551 and *M. smegmatis* mc (b)155 strains, were employed in all investigations. The lethal mechanisms suggested that AC2P36 and AC2P20 depleted free thiol pools and increased intracellular ROS. Also, it has been found that AC2P20 depletes fewer free thiols than AC2P36, despite the fact that it causes more intracellular ROS to accumulate. While both of them seemed to target *M. tb* free thiols, it is possible that the processes they follow are distinct from one another. Figure 18 demonstrated the no-effect-on-time dependence killing when neutral conditions were employed, whereas at pH 5.7, 100-fold reduction was indicated in comparison with DMSO control as far as viability is concerned. Phenyl-dioxypyrimidine release may also target the secondary undiscovered *M. tb* physiological mechanism, which may explain the larger ROS rise that was reported in comparison to AC2P36.

In light of this recent research, thiol homeostasis as an alternate method to eliminate *M. tb* at acidic pH has been further validated. Auranofin and other chemotypes that work in an indirect manner are usually most promising. Compounds similar to AC2P20 or AC2P36 could, however, be developed into prodrugs that are activated by a *M. tb*-specific enzyme in order to selectively release the thiol-reactive warhead within the bacterial cell. Furthermore, isolating the resistant mutants was not possible for AC2P20 and AC2P36.

**SCHEME 42:**
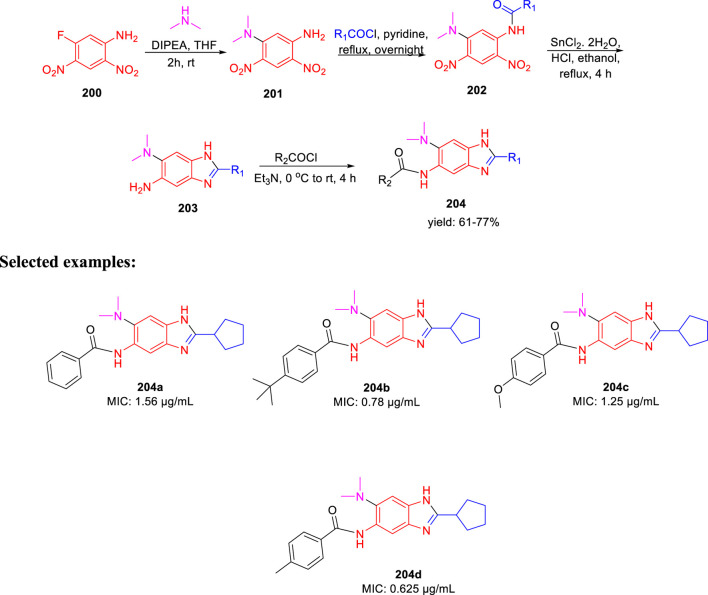
Synthetic route to 2,5,6-trisubstituted benzimidazoles *via* amide bond formation.

Ojima *et al.* accomplished the synthesis of 2,5,6-trisubstituted benzimidazole moieties and assessed their antitubercular activity by targeting *Mtb*-FtsZ (Haranahalli et al., 2021). As depicted in Scheme 42, compound **200** was reacted with secondary amine in the presence of DIPEA to form intermediate **201**. In the next step, acylation of intermediate **201** produced **202**, which further underwent reduction followed by cyclization in the presence of SnCl_2_.H_2_O to form 2,5,6-trisubstituted benzimidazoles **203** (Kumar et al., 2011; Awasthi et al., 2013). In the last step, acylation of compound **203** achieved the desired moiety **204**. The initial SAR analysis of this library revealed a preference for substituents with cyclopentyl, pent-3-yl, isopropyl, and benzyl groups in their second positions. In the initial screening, none of the compounds with a phenyl or 2-furyl substituent in position 2 had significant activity. When tested with the resynthesized compound, one compound showed poor inhibition activity of cell growth (MIC >10 µg/ml), despite one hit with a thien-2-yl substituent at position 2. According to these findings, at position 2, a Sp3 hybridized carbon is preferred over a Sp2 carbon. Some of the synthesized compounds exhibited exceptional MIC values toward H37Rv strain such as 0.78 and 0.625 µg/ml for **204b** and **204d**, respectively.

**FIGURE 19:**
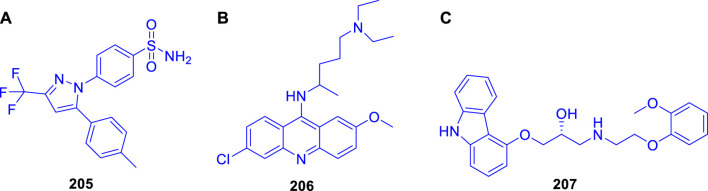
**(A)** Structures of celecoxib **205**, **(B)** Structure of quinacrine **206**, and **(C)** Structure of carvedilol **207**.

Cheng *et al.* reported the virtual screening of nonbenzofuran inhibitors against *Mycobacterium tuberculosis* Pks13-TE for anti-TB phenotypic discovery based on their structures (Zhao et al., 2021). It seemed that only celecoxib **205** and quinacrine **206** showed inhibitory effects against *M.* tuberculosis strain H37Ra (Figure 19). Pks13-TE soaked with Tam16 is the optimum structure to undertake virtual screening, according to previous studies. Tam16 significantly impairs mycolic acid’s ability to bind to the active site and exert its inhibitory effect. This was the first investigation to use Pks13-TE for *in silico* studies based on Glide-SP docking. *In vitro* antimicrobial phenotypic activity tests were also performed to look for novel antitubercular drugs using innovative scaffolds to overcome the restriction of the benzofuran core. Antituberculosis activity with an MIC value of 32 μg/ml was seen in the mild antituberculosis compound carvedilol **207**, whereas the remaining hit compounds had an MIC value of 64 μg/ml. To screen the FDA database for antitubercular medicines, a dependable structure-based digital screening method was developed. Three long-established medications, carvedilol, celecoxib, and quinacrine, were found to have antitubercular action, comparable stability, and binding mechanisms with co-crystal ligand Tam16, which was supported by the simulation data. Excellent druggability and low toxicity of these compounds can be useful for promoting the development of antitubercular agents in future.

**FIGURE 20:**
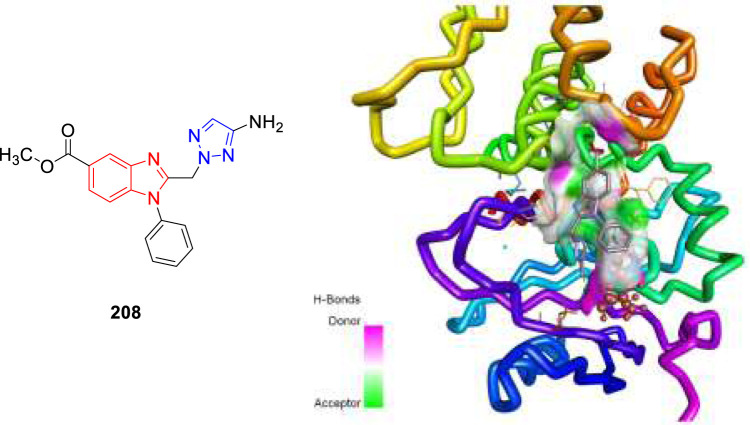
Binding pattern of compound **208** with PrpR protein of *Mycobacterium tuberculosis.*

In 2021, our group identified a unique class of biheterocyclic molecules to target tuberculosis through a computational study (Rajasekhar et al., 2021). In this regard, we have designed around 20 molecules of substituted benzimidazolyl triazoles to target the *Mycobacterium tuberculosis* protein PrpR, as shown in Figure 20.

It is interesting to observe that in regulating the activity of PrpR protein of *Mycobacterium tuberculosis*, a 4Fe4S cluster binding site and CoA binding site play key roles (Tang et al., 2019). For PrpR to display its role in the methyl citrate cycle (MCC), which involves the elimination of propionyl-CoA a cholesterol degradation product from the system well, there are numerous transcription factors required to be activated. Our understanding is that the designed molecules could inhibit the PrpR protein by ruling out the binding of CoA to its active site, which is further synchronized by the [4Fe4S] clusters binding in the neighboring chain. The mentioned procedure resulted in the several structural deformations that might play an important role in influencing the functions of MCC. We have performed the AutoDock Vina and Glide module for the molecular docking investigation of designed compounds. Based on the Prime-MM/GBSA and the QikProp module, the binding energies and physiochemical properties of the designed molecules were evaluated, respectively. Additionally, a machine learning-based algorithm was used to rank the aforementioned compounds by predicting and evaluating the inhibitory effects of scaffolds. Subsequently, one compound **208** was then subjected to molecular dynamics simulation study to validate the binding characteristics of compounds against PrpR of *Mycobacterium tuberculosis*.

This year also we have designed and computationally evaluated the antitubercular property of substituted thiazolidines, a prominent five-membered N–S heterocycles targeting the PrpR protein of *Mycobacterium tuberculosis* (Rajasekhar et al., 2022).

We have performed the AutoDock Vina and Glide module for the molecular docking investigation of all 17 designed compounds. Based on the Prime-MM/GBSA and the ADMET study, the binding energies and physiochemical properties of the designed molecules were evaluated, and it was found that compound **209** exhibits better binding scores compared to standard drug isoniazid. MD simulation studies of 20 ns validate the structural modifications and dependability of the binding affinities of the top-hit compound **209**.

## Conclusion

**FIGURE 21:**
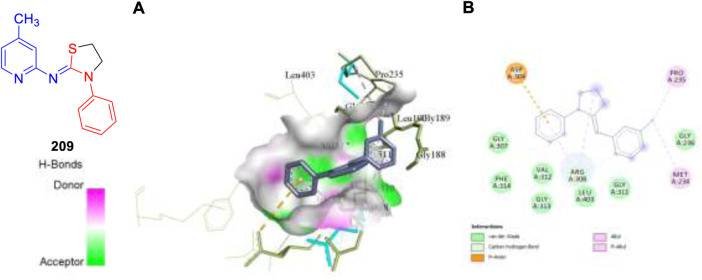
Molecular docking visualization and its binding pattern of compound 209. **(A)** Binding of compound **209** at the central cavity. **(B)** 2D depiction and nature of binding interaction involved.

Pharmaceutical companies have recently faced several roadblocks owing to an enhanced focus on complicated diseases without knowing their biology, followed by a highly competitive landscape from emerging new infectious diseases along with the pricing pressures from patients and buyers. At this juncture, drug discovery scientists along with highly motivated synthetic organic chemists can change the complete scenario by selecting the relevant targets for human diseases and identifying the molecules along with their most feasible synthetic routes. Furthermore, by investing huge resources in synthetic chemistry and chemical technology field, we can advance the area to a position of exploration of chemical moieties in an unimpeded way. Furthermore, it is to be noted that the tremendous progress made in this field of antitubercular drug discovery until now has been possible because of a close coordination between the industry and academia in the last few years. Despite great efforts, only a small number of the hundred powerful compounds found as anti-TB medications were able to enter the clinical stage. There have only been three new medications for tuberculosis in the past 50 years. This may be a result of the difficulties in discovering therapeutic candidates that are both effective and benign, have an appropriate PK profile, and can treat multidrug-resistant TB using innovative mechanisms. The synthetic routes described in this review article for the synthesis of antitubercular drug candidates will pave the way for inventing new medicines for the betterment of patient lives throughout the world in the future (Figure 21).

## Abbreviations

WHO, World Health Organization; HIV, human immunodeficiency virus; FDA, Food and Drug Administration; INH, isoniazid; MIC, minimum inhibitory concentration; SAR, structure–activity relationship; *M.tb*, *Mycobacterium tuberculosis*; RIF, rifampicin; IC_50_, inhibitory concentration; LD_50_, lethal dose; CC_50_, cytotoxic concentration; DNA, deoxyribonucleic acid; and ATP, adenosine triphosphate.
